# Characterization of a Sulfhydryl Oxidase From *Plasmodium berghei* as a Target for Blocking Parasite Transmission

**DOI:** 10.3389/fcimb.2020.00311

**Published:** 2020-06-26

**Authors:** Wenqi Zheng, Fei Liu, Feng Du, Fan Yang, Xu Kou, Yiwen He, Hui Feng, Qi Fan, Enjie Luo, Hui Min, Jun Miao, Liwang Cui, Yaming Cao

**Affiliations:** ^1^Department of Immunology, College of Basic Medical Sciences, China Medical University, Shenyang, China; ^2^Department of Clinical Laboratory, Affiliated Hospital of Inner Mongolian Medical University, Hohhot, China; ^3^Department of Pathogen Biology, College of Basic Medical Sciences, China Medical University, Shenyang, China; ^4^Department of Animal Quarantine, College of Animal Husbandry and Veterinary Sciences, Liaoning Medical University, Jinzhou, China; ^5^Dalian Institute of Biotechnology, Dalian, China; ^6^Department of Internal Medicine, Morsani College of Medicine, University of South Florida, Tampa, FL, United States

**Keywords:** malaria, *Plasmodium berghei*, quiescin sulfhydryl oxidase, sexual development, transmission-blocking vaccine

## Abstract

Quiescin sulfhydryl oxidase (QSOX), present in a wide variety of eukaryotic species, catalyzes the insertion of disulfide bonds into unfolded, reduced proteins. Here we characterized the QSOX protein from the rodent malaria parasite *Plasmodium berghei* (PbQSOX), which is conserved in all sequenced malaria parasite species. The PbQSOX protein was not expressed in asexual erythrocytic stages, but was most abundantly expressed in ookinetes. Indirect immunofluorescence assays revealed PbQSOX was not only localized in cytoplasm of gametocytes, gametes and ookinetes, but also expressed on the surface of gametes and ookinetes. Western blot identified extracellular presence of PbQSOX in the culture medium of ookinetes suggestive of secretion. *Pbqsox* deletion (Δ*pbqsox*) did not affect asexual intraerythrocytic development, but reduced exflagellation of male gametocytes as well as formation and maturation of ookinetes. *Pbqsox* deletion also led to a significant increase in the reduced thiol groups of ookinete surface proteins, suggesting that it may play a role in maintaining the integrity of disulfide bonds of surface proteins, which might be needed for ookinete development. Mosquitoes that fed on Δ*pbqsox*-infected mice showed a significant reduction in ookinete and oocyst numbers compared to those fed on wild-type parasite-infected mice. Further, both polyclonal mouse antisera and a monoclonal antibody against the recombinant PbQSOX exhibited substantial transmission-blocking activities in *in vitro* and mosquito feeding assays, suggesting QSOX is a potential target for blocking parasite transmission.

## Introduction

Malaria is caused by protozoan parasites of the genus *Plasmodium* and transmitted via female *Anopheles* mosquitoes. Globally, an estimated 3.3 billion people are at risk of infection. According to World Malaria Report 2019, the global progress against malaria has stalled as the number of malaria cases rose from 217 million in 2016 to 228 million in 2018 (WHO, 2019). The emergence of drug-resistant parasites and insecticide-resistant *Anopheles* are major limiting factors for malaria elimination, and calls for integrated approaches (Hemingway et al., 2016; Menard and Dondorp, 2017). One potential strategy involves the development of effective malaria vaccines (Genton, 2008). Among the vaccines targeting different parasite stages, transmission-blocking vaccines (TBVs) target sexual- and mosquito-stage parasites (i.e., gametocyte, gamete, zygote, and ookinete) as well as mosquito gut proteins (Wu et al., 2015; Kumar and Tolia, 2019).

The working principle of TBVs is that TBVs induce transmission-blocking (TB) antibodies in humans, which will arrest subsequent parasite development in the mosquito midgut, thus interrupting the transmission of the parasites to the vectors (Wu et al., 2015; Delves et al., 2018). Transmission of the malaria parasites to the vector is initiated by the sexual stage precursor cells, the gametocytes. Once gametocytes are ingested by a mosquito, gametogenesis is accomplished in 15–20 min, and the resultant male and female gametes will mate to form zygotes. Subsequent development from a zygote to a motile ookinete must be accomplished within 24 h so that the parasite can escape the hostile environment in the blood bolus. The gamete–ookinete stages are extracellular and are exposed to the mosquito-derived digestive enzymes and the resultant cytotoxic byproducts, as well as the immune responses from the human hosts (Sinden, 2002). To adapt to these environmental changes in the blood meal inside the mosquito midgut, the malaria parasite produces antioxidant proteins such as thioredoxin-1, peroxiredoxin-1 and 1-Cys peroxiredoxin-1, to ensure survival and escape of the ookinete (Turturice et al., 2013). However, it is not known whether other antioxidant proteins are involved in this process or how the ookinete deals with the oxidative damage of its surface proteins.

Quiescin sulfhydryl oxidase (QSOX) family enzymes, found in eukaryotes except fungi, specifically catalyze the direct and facile introduction of disulfide bonds into unfolded, reduced proteins with the reduction of a molecular oxygen, generating hydrogen peroxide: 2RSH+O_2_ → RS-SR+H_2_O_2_ (Haque et al., 2012; Limor-Waisberg et al., 2013). Two moieties of the enzyme carry out the tandem actions of substrate oxidation and electron transfer: thioredoxin-fold (Trx) domain and a sulfhydral oxidase module related to the Erv/ALR enzyme family. A helix-rich region between the Trx and Erv/ALR domains adopts a structural fold similar to the Erv domain, and is thus known as the pseudo-Erv domain (ψErv) (Alon et al., 2010). Interestingly, metazoan QSOX enzymes have a second Trx-fold domain (Trx-2) between the active Trx domain and the ψErv domain, whereas this Trx-2 domain is absent in plant and protist QSOXs (Kodali and Thorpe, 2010; Haque et al., 2012; Limor-Waisberg et al., 2013). While the functional significance of QSOXs has been increasingly appreciated, recent studies have revealed that elevated human QSOX is strongly correlated with certain diseases including pancreatic cancer (Katchman et al., 2011), heart failure (Mebazaa et al., 2012), breast cancer (Soloviev et al., 2013; Poillet et al., 2014), and prostate tumorigenesis (Song et al., 2009).

In looking for additional antioxidant proteins, we paid attention to the QSOX family because there is a growing body of evidence showing that QSOXs are widely present in eukaryotes and may play essential roles in parasite growth and host–parasite interactions (Haque et al., 2012). The QSOX enzymes that catalyze the thiol redox reaction possess conserved as well as unique domains, making them worthwhile targets for new therapeutic development. Here we identified a QSOX member in all malaria parasite genomes and characterized QSOX in the rodent malaria parasite *Plasmodium berghei* (PbQSOX). We discovered that PbQSOX is required for parasite sexual development, especially for ookinete maturation. In addition, antibodies against PbQSOX showed obvious TB activities, indicating that PbQSOX may be a potential candidate for TB drug and vaccine development.

## Materials and Methods

### Mice, Parasites, and Mosquitoes

Six- to eight-week-old female BALB/c mice were used for all experiments. *P. berghei* (ANKA strain 2.34) and all experimental lines were maintained in female BALB/c mice by serial mechanical passage (up to a maximum of eight passages) and used for challenge infection as described elsewhere (Blagborough and Sinden, 2009). Mouse infection was typically done by intraperitoneally (i.p.) injecting 5 × 10^6^ *P. berghei*-infected red blood cells (iRBCs). Adult *Anopheles stephensi* (Hor strain) mosquitoes were maintained on a 10% (w/v) glucose solution at 25°C and 50–80% relative humidity with a 12-h light–dark cycle in an insectary. Animal use was conducted according to the guidelines of The Animal Usage Committee of China Medical University.

### Sequence Analysis

The putative *pbqsox* amino acid sequence and its orthologs in other *Plasmodium* species in PlasmoDB (www.plasmodb.org) were used to BLAST search GenBank. Homologous genes in model organisms were retrieved and aligned by ClustalW. A cladogram of the full-length QSOX proteins was constructed using the maximum-likelihood method as implemented in MEGA5 (Tamura et al., 2011). The secondary structure of PbQSOX was predicted by PSIPRED (http://bioinf.cs.ucl.ac.uk/psipred/). Signal peptide and protein domain organization was determined using the SMART program (http://smart.embl-heidelberg.de/).

### Expression and Purification of Recombinant PbQSOX (rPbQSOX)

For the expression of rPbQSOX, a *pbqsox* fragment encoding amino acids 24–516 without the predicted signal peptide was amplified from the *P. berghei* genomic DNA using forward primer *pbqsox*-F containing a *Bam*HI site and an enterokinase cleavage sequence (GACGACGACGACAAG) (Zhou et al., 2014), and reverse primer *pbqsox*-R containing a *Hin*dIII site (Table S1) and cloned into pET30a (+) vector. The final pET-30a (+)-*pbqsox* vector carried an N-terminal His•Tag®/thrombin/S•Tag™/ enterokinase sequence and the *pbqsox* fragment. The rPbQSOX was expressed in *Escherichia coli* BL-21 strain after induction with 1 mM isopropyl-β-D-thiogalactopyranoside at 20°C for 12 h. Proteins were purified using Ni-NTA His•Bind Superflow (Novagen) under native conditions. After the final wash with 20 mM imidazole in 300 mM NaCl and 50 mM sodium phosphate buffer (pH 8.0), rPbQSOX was eluted with 250 mM imidazole in 300 mM NaCl and 50 mM sodium phosphate buffer (pH 8.0). The fractions containing rPbQSOX were extensively desalted in 0.1 M phosphate-buffered saline (PBS, pH 7.4) overnight at 4°C. Then the fusion rPbQSOX protein was digested by the recombinant enterokinase (Solarbio) at 25°C for 16 h to remove the tags. The mixture was further purified by Ni-NTA His•Bind Superflow to remove the His/S tag. The effluent containing liberated rPbQSOX was analyzed by 10% SDS-PAGE. A recombinant glutathione S-transferase (rGST) protein was expressed using the empty vector pGEX-4T-1 and used as negative controls for activity assay and immunization.

### PbQSOX Activity Assay

The sulfhydral oxidase activity of rPbQSOX was determined at 25°C in 50 mM potassium phosphate buffer and 1 mM EDTA at pH 7.5 as described elsewhere (Raje et al., 2002; Zheng et al., 2012). The molar concentration of the enzyme was determined according to the molecular weight determined using the molar extinction coefficient from the ExPASy proteomics server (http://www.expasy.org/). Briefly, 500 nM of purified rPbQSOX or rGST (negative control) was assayed in a final assay mixture containing 1.4 μM horseradish peroxidase (HRP), 1 mM homovanillic acid (HVA) and 5.7 mM Tris [2-carboxyethyl] phosphine (TCEP) or 5 mM dithiothreitol (DTT) (Sigma). In this assay, the oxidase activity was determined as the fluorescence intensity of the HVA dimer, which was formed from HRP-mediated HVA oxidation by H_2_O_2_. H_2_O_2_ is produced when oxygen is reduced by QSOX using TECP or DTT as the substrate (Raje et al., 2002). Enzyme activity data were obtained by monitoring the fluorescence at 360 nm excitation and 485 nm emission at 10 min.

### Purification of Different Stages of the Parasite

To purify schizonts, infected blood taken from mice on day 4 post infection (p.i.) were placed in a blood-stage culture medium [RPMI 1640 containing 20% (v/v) fetal bovine serum (FBS), 50 mg/L penicillin and streptomycin, 50 ml culture medium/0.5 ml blood] and incubated for 16 h at 37°C with shaking at 100 rpm. A Giemsa-stained blood smear from the culture was prepared and the relative abundance of schizonts determined. If appropriate, the culture was fractionated on 55% (v/v) Nycodenz–RPMI 1640 culture medium cushion. Gametocytes were purified according to a previous study (Beetsma et al., 1998) with modifications. Phenylhydrazine-treated mice were infected with *P. berghei*, and on day 4 p.i. mice were treated with 20 mg/L sulfadiazine (Sigma) in drinking water for 2 days. Blood was collected in blood-stage culture medium pre-warmed at 37°C to avoid premature activation of gametocytes, and the blood suspension was loaded on top of a 48% (v/v) Nycodenz/blood-stage culture medium cushion and centrifuged at 1,300 × g for 30 min without brake. The interface was recovered and washed twice in 10 ml of RPMI 1640 before activation for gamete formation. For ookinete culture, parasitemia was allowed to reach 1–3% and mice were bled by cardiac puncture under terminal anesthesia on day 3 p.i. as described (Sinden et al., 1985). Blood was passed through a CF11 cellulose powder (Whatman) column to deplete white blood cells. The eluate was diluted 1:10 with complete ookinete medium [RPMI 1640 containing 50 mg/L penicillin, 50 mg/L streptomycin, 100 mg/L neomycin, 20% (v/v) FBS and 1 mg/L heparin, pH 8.3] in a flask to a maximum depth of 1 cm and kept at 19°C for 24 h, after which the culture was checked for the presence of ookinetes by Giemsa staining. Cultured ookinetes were loaded on a 62% (v/v) Nycodenz/ookinete culture medium cushion and centrifuged at 1,300 × g for 30 min without brake. Ookinetes at the interface were recovered and washed twice in 10 ml of PBS (pH 7.4).

### Quantitative Reverse Transcription-Polymerase Chain Reaction (qRT-PCR)

Total RNA was isolated from purified parasites using an RNA purification kit (Qiagen). cDNA was synthesized from 1 μg of total RNA using an RNA-to-cDNA kit (Takara). *Pbqsox* expression was determined by qRT-PCR using primers Pbqsox1 and Pbqsox2 (Table S1). qRT-PCR reactions consisted of 2 μl cDNA, 10 μl SYBR Green fast master mix (ThermoFisher), 0.5 μl each of the forward and reverse primers and 7 μl RNase-free water. Analysis was conducted using a 7500 Fast PCR System (ThermoFisher) with the following conditions: initial denaturation at 95°C for 20 s, followed by 40 cycles of 95°C for 5 s and 60°C for 30 s. The expression of *hsp70* (PBANKA_081890) was used as the internal reference with primers Hsp70F and Hsp70R (Table S1). Relative quantification of *pbqsox* expression was assessed using the ΔΔC_t_ method (Livak and Schmittgen, 2001).

### Animal Immunization and Antibody Production

To obtain specific immune sera, purified rPbQSOX was mixed with complete Freund's adjuvant and used to subcutaneously immunize BALB/c mice (50 μg/mouse). Two booster immunizations of 25 μg of protein emulsified in incomplete Freund's adjuvant were done at 2-weeks intervals. Ten days after the last immunization, blood was collected from the mice by cardiac puncture and allowed to clot at room temperature to obtain the antisera.

Anti-rPbQSOX monoclonal antibody (mAb) was produced using spleen cells obtained from BALB/c mice immunized with rPbQSOX as described above and fused with Sp2/0-Ag14 myeloma cells (Kohler and Milstein, 1975). Hybridoma cells were generated by the polyethylene glycol method, selected in the hypoxanthine-aminopterin-thymidine medium, and screened by indirect antibody capture enzyme-linked immunosorbent assay (ELISA). The IgG fractions were prepared by ammonium sulfate precipitation and purified on a Protein A column. The mAb isotype was determined using the SBA Clonotyping™ System-HRP (Southern Biotechnology Associates) according to the manufacturer's instructions.

### Western Blot

Purified schizonts, gametocytes and ookinetes were treated with 0.15% saponin to lyse erythrocytes and parasites were collected by centrifugation and washed once with PBS. Parasites were lysed in PBS containing 1% Triton X-100, 2% SDS, and protease inhibitors (Volkmann et al., 2012) for 30 min at room temperature. Equal amounts of the parasite lysates (10 μg/per lane) were separated on a 10% SDS-PAGE gel under reducing conditions. Proteins were transferred to a 0.22 μm PVDF membrane (Bio-Rad). The membrane was blocked with 5% skim milk in Tris-buffered saline (TBS) at 4°C for 12 h and then incubated with anti-rPbQSOX antisera diluted at 1:500 or anti-PbQSOX mAb diluted at 1:1,000 in TBS containing 0.1% Tween 20 (TBST) for 3 h. The mouse anti-PbHsp70 sera (1:500) were used to monitor protein loading. After three washes with TBST, the membrane was incubated for 2 h with HRP-conjugated goat anti-mouse IgG antibodies (Invitrogen) diluted 1:10,000 in TBST. After three washes with TBST, the proteins on the blot were visualized with a Pierce ECL Western Blotting Kit (ThermoFisher).

### Indirect Immunofluorescence Assay (IFA)

Cells were washed once in PBS and fixed with 4% paraformaldehyde and 0.0075% glutaraldehyde (Sigma) in PBS for 30 min at room temperature (Tonkin et al., 2004). Fixed cells were washed once in PBS and then permeabilized with or without 0.1% Triton X-100 in PBS for 10 min. Cells were rinsed with 50 mM glycine in PBS and blocked with PBS containing 3% skim milk for 1 h at 37°C. Cells were incubated with mouse anti-rPbQSOX antisera (1:500) or anti-rPbQSOX mAb (1:500), or anti-Pbs21 mAb (1:500) in PBS containing 3% skim milk at 37°C for 1 h and washed three times with PBS. The parasites without permeabilization were then treated with 0.1% Triton X-100 and blocked with 3% bovine serum albumin/PBS for 60 min. After that, all parasites were incubated with rabbit antisera against PbMSP1, Pbg377, α-tubulin and PSOP25 as stage-specific markers for schizonts, female gametocytes/gametes, male gametocytes/gametes, and ookinetes, respectively (Liu et al., 2019). After the cells were washed thrice with PBS, Alexa-488 conjugated goat anti-mouse IgG secondary antibodies (1:500; Invitrogen) and Alexa-555 conjugated goat anti-rabbit IgG secondary antibodies (1:500; Abcam) were added and incubated for 1 h. Cells were mounted with Hoechst 33258 (1:1,000; Invitrogen). Negative controls were wild-type (WT) *P. berghei* ookinetes that were incubated with the anti-rGST sera or with the secondary antibodies only. WT ookinetes probed with the anti-Pbs21 mAb and anti-PSOP25 serum served as the positive controls. Parasites were visualized on a Nikon C2 fluorescence confocal laser scanning microscope (Nikon, Japan).

### Generation of the *Pbqsox* Knockout Lines

To knock out *pbqsox*, an 824-bp upstream fragment containing the 5′ UTR was amplified from *P. berghei* genomic DNA using primers 5UTR-F and 5UTR-R (Table S1), and cloned in the plasmid PL0034 at the *Hin*dIII and *Pst*I sites upstream of the h*dhfr* cassette. Similarly, an 805-bp downstream fragment containing the 3′ UTR was amplified using primers 3UTR-F and 3UTR-R (Table S1), and inserted at the *Xho*I and *Eco*RI sites downstream of the h*dhfr* cassette. This would replace the *pbqsox* protein-coding region with the h*dhfr* expression cassette which confers resistance to pyrimethamine. The plasmid was linearized by *Hin*dIII/*Eco*RI digestion and transfected by electroporation into purified *P. berghei* schizonts (Janse et al., 2006). In two independent transfection experiments, the complete parasite suspension was injected intravenously via the tail vein into two mice. Starting at 24 h after injection of the transfected parasites, mice were treated with pyrimethamine (Sigma) in drinking water at 70 μg/ml for a period of 3–4 days. After parasite cloning by limiting dilution, the *pbqsox* deletion (Δ*pbqsox*) line was confirmed by integration-specific PCR (Table S1). The Δ*pbqsox* line was also confirmed by Western blot and IFA. For Western blot, WT and Δ*pbqsox* ookinetes were purified and incubated with the anti-rPbQSOX sera and anti-Hsp70 sera as described above. For IFA, Δ*pbqsox* ookinetes were incubated with anti-rPbQSOX sera after membrane permeabilization with Triton X-100.

### Phenotypic Analysis of the Δ*pbqsox* Line

To determine whether deletion of *pbqsox* affects parasite growth, five mice were inoculated i.p. with 0.2 ml of infected blood containing either 5 × 10^6^ WT or Δ*pbqsox* parasites. Asexual parasitemia was monitored from day 3 to day 7 p.i. using Giemsa-stained blood films. To evaluate the effect on gametocytogenesis and gametocyte activation, BALB/c mice were injected i.p. with 0.2 ml of 6 mg/ml phenylhydrazine in 0.9% NaCl 3 days prior to infection. Mice were inoculated i.p. with 0.2 ml of infected blood containing 5 × 10^6^ iRBCs. Three days after infection, gametocytemia and gametocyte sex ratio were assessed by Giemsa-stained blood smears (Lal et al., 2009). Exflagellation centers of male gametocytes and formation of macrogametes were quantified as described (Tewari et al., 2010; van Dijk et al., 2010). Briefly, a volume (close to 10 μl) of gametocyte-infected blood adjusted to contain equal amounts of mature gametocytes based on the mature gametocyte count was obtained from the tail vein of each mouse and mixed immediately with ookinete culture medium in a final volume of 50 μl. The mixture was placed under a Vaseline-coated cover slip at 25°C and 15 min later exflagellation centers (male gamete interacting with RBCs) were counted over the next 10 min under a phase contrast microscope at 400× magnification. To count macrogametes, 10 μl of gametocyte-infected blood were mixed with ookinete culture medium at 25°C for 15 min. Then the macrogametes were labeled with anti-Pbs21 mAb (1:500) and Alexa-488-conjugated anti-mouse IgG antibodies (1:500) without membrane permeabilization. The culture was continued at 19°C for 24 h, harvested and labeled with mouse anti-Pbs21 mAb (1:500) and Alexa-488-conjugated anti-mouse IgG antibodies (1:500). Detailed analysis of ookinete differentiation was performed as described (Janse et al., 1985). Macrogametes, zygotes and different stages of ookinetes were counted under a fluorescence microscope at 1,000× magnification in 20 fields. The proportions of different cell shapes were calculated as the number of zygotes or ookinetes/(total number of macrogametes, zygotes and different stage ookinetes) ×100% as described previously (Reininger et al., 2005).

For mosquito feeding, 4 day-old female *An. stephensi* mosquitoes were starved for 12 h and then allowed to feed on phenylhydrazine-treated mice infected with either WT or Δ*pbqsox* parasites for 30 min. Unfed mosquitoes were removed and engorged mosquitoes were maintained at 19–22°C and 50–80% relative humidity. The ookinete formation in the blood meal was analyzed at 24 h after blood feeding (Volkmann et al., 2012). For oocyst counts, midguts were dissected on day 10 and stained with 0.5% mercurochrome (Sigma) to determine the prevalence (proportion of infected mosquitoes) and intensity (number of oocysts per positive midgut) of infection (Usui et al., 2011).

### Quantification of PbQSOX in Ookinete Culture Medium

Ookinetes were cultured as described above and the culture medium was collected by centrifugation at 1,300× g for 10 min. To detect the presence of PbQSOX, culture medium (50 μl/lane) was separated on a 10% SDS-PAGE gel under reducing conditions and Western blots were performed as described above with the anti-rPbQSOX mAb (1:1,000 dilution). To quantify PbQSOX in the ookinete culture medium, a standard ELISA curve with purified rPbQSOX at 0.1, 0.2, 0.4, 0.6, 0.8, 1, and 1.6 μg/well was constructed. Then 50 μl of serially diluted ookinete culture medium (from a known number of the ookinetes) were added to each well of a Nunc MaxiSorp® flat-bottom 96-well plate. After coating of plate at 4°C for 12 h, the plate was sequentially incubated with anti-rPbQSOX mAb (1:1,000) and HRP-conjugated goat anti-mouse IgG antibodies (1:2,000, Invitrogen). Then the PbQSOX content of the WT ookinete culture medium was calculated according to the standard curve.

### Quantification of Thiol Groups on Δ*pbqsox* Ookinetes by ThioGlo Staining

To quantify the amount of thiol groups on the ookinetes, 100 ookinetes from the WT and Δ*pbqsox* parasites were washed with PBS (pH 7.4) for four times. Then the samples were incubated with 6 μM of the ThioGlo fluorescent probe IV (Calbiochem) in the dark for 30 min at room temperature. The reaction was terminated by the addition of 2 μl of 2 M HCl, and the fluorescence was measured by using an ELISA plate reader with excitation at 400 nm and emission at 465 nm. Background fluorescence was subtracted from all fields (Ilani et al., 2013). The amount of thiol groups on both WT and Δ*pbqsox* ookinetes were compared.

### *In vitro* Ookinete Conversion Inhibition Assay

Mice pre-treated with phenylhydrazine were infected as described above. On day 3 p.i., parasitemia was determined and exflagellation of male gametocytes was checked. Ten μl of infected blood were taken from each mouse and added to 90 μl ookinete medium containing anti-rPbQSOX antisera or anti-rGST sera as the control at final dilutions of 1:5, 1:10, and 1:50. Additionally, anti-rPbQSOX mAb was added to the ookinete culture at 10, 5, and 1 μg/100 μl of ookinete culture, respectively. The culture without mAb was used as a negative control. Ookinete cultures were incubated at 19°C for 24 h and the number of macrogametes, zygotes, retorts and ookinetes were counted as described above. Ookinete conversion rate were calculated as the number of ookinetes/the total number of (macrogametes, zygotes, retorts and ookinetes) ×100%.

### *In vivo* TB Activity

For *in vivo* studies, three mice were immunized with the rPbQSOX as described above. Three mice in the control group were immunized with the rGST protein. Ten days after the last immunization, six immunized mice were infected with the WT *P. berghei* iRBCs to assess TB activity by the direct mosquito feeding assay. For the antibody transfer experiment, three mice were injected intravenously with either 150 μg of anti-rPbQSOX mAb/mouse or equal volume of PBS as control 1 h before mosquito feeding. Approximately 30 mosquitoes were dissected 10 days after feeding to determine the prevalence and intensity of infection.

### Statistical Analysis

Statistical comparison between groups was performed with the GraphPad Prism 6.0 software. Parasitemia, gametocytemia, the amount of PbQSOX secreted by ookinetes and thiol contents were analyzed by the Student's *t* test and ANOVA. The number of exflagellation centers, macrogamete numbers and the intensity of infection (oocysts/midgut) were analyzed by the Mann–Whitney *U* test, while the proportions of cell shapes and ookinete conversion rate were analyzed by the Chi-square test. The prevalence of infection was analyzed by the Fisher's exact test using SPSS version 21.0. *P* < 0.05 was considered statistically significant.

## Results

### Bioinformatic Analysis Identifies a Conserved QSOX Gene in *Plasmodium*

**Figure 1:**
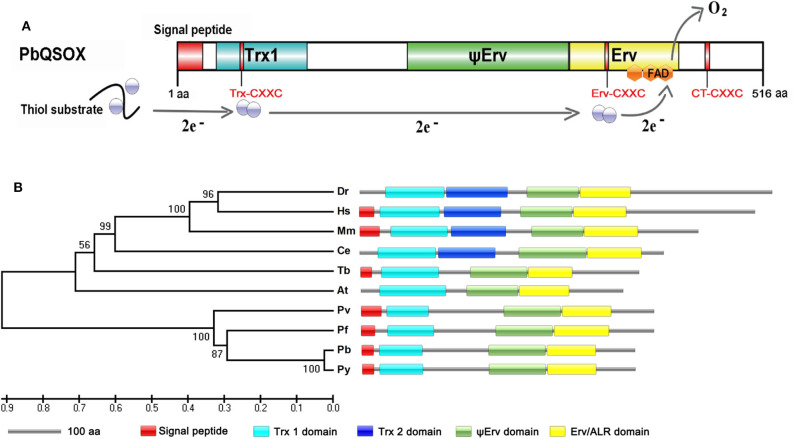
Identification and phylogenetic analysis of PbQSOX. **(A)** Domain organization of PbQSOX protein and schematic representation of the electron transfer pathway through PbQSOX domains. Trx1, ψErv, and Erv/ALR domains are highlighted in blue, green and yellow, respectively. Within the oxidoreductase module of QSOX, two electrons are accepted from the substrate by the Trx-CXXC motif. From the Trx1 domain, the electrons are transferred to the Erv-CXXC motif at first, then to the FAD cofactor. Ultimately, two electrons are transferred to molecular oxygen through the FAD cofactor. **(B)** A maximum likelihood tree inferred from QSOXs in different species: *Arabidopsis thaliana* (At), *Caenorhabditis elegans* (Ce), *Danio rerio* (Dr), *Homo sapiens* (Hs), *Mus musculus* (Mm), *Plasmodium berghei* (Pb), *Plasmodium falciparum* (Pf), *Plasmodium vivax* (Pv), *Plasmodium yoelii* (Py), and *Trypanosoma brucei* (Tb). Protein domain architectures predicted with the SMART program are shown.

Search of the PlasmoDB for proteins involved in disulfide bond formation identified a QSOX-like protein in all sequenced *Plasmodium* genomes. This putative sulfhydryl oxidase protein in *P. berghei* (PBANKA_145540) encodes 516 amino acids, with a predicted molecular weight of 61.5 kDa. Analysis of the domain structures by SMART showed that the predicted protein contains a signal peptide, a highly conserved N-terminal Trx1 domain and C-terminal Erv/ALR domain, which is typical of QSOX enzymes (Figure 1A). Phylogenetic comparison with homologs in model organisms showed that all *Plasmodium* homologs formed a separate clade, most closely related to the plant *Arabidopsis thaliana* QSOX (Figure 1B). From the sequence alignment and predicted domains (Figure 1B and Figure S1A), *Plasmodium* QSOX-like proteins are different from the metazoan homologs in that it lacks the Trx2 domain completely. This feature closely resembles QSOX proteins in plants and protozoan parasites (Figure 1B). The *Plasmodium* QSOX-like proteins have all three conserved CXXC motifs. Within the Trx1 domain, the most prevalent redox active Trx-CXXC motif is CGHC, whereas the corresponding PbQSOX sequence is CPAC, which is the same as in *Arabidopsis* (Figure S1). The Erv/ALR domain Erv-CXXC motif serves to communicate with the Trx-CXXC and interacts with the flavin adenine dinucleotide (FAD) co-factor (Haque et al., 2012), and this motif in PbQSOX is CRNC (Figure S1A). In addition, a C-terminal CXXC motif (CT-CXXC) is also highly retained in QSOX family enzymes (Figure S1A). Thus, the putative sulfhydryl oxidase protein in *P. berghei* has all these conserved features of the QSOX family, and is thus designated as the *P. berghei* quiescin sulfhydryl oxidase (PbQSOX).

### The Recombinant PbQSOX Possesses Thiol Oxidase Activity

**Figure 2:**
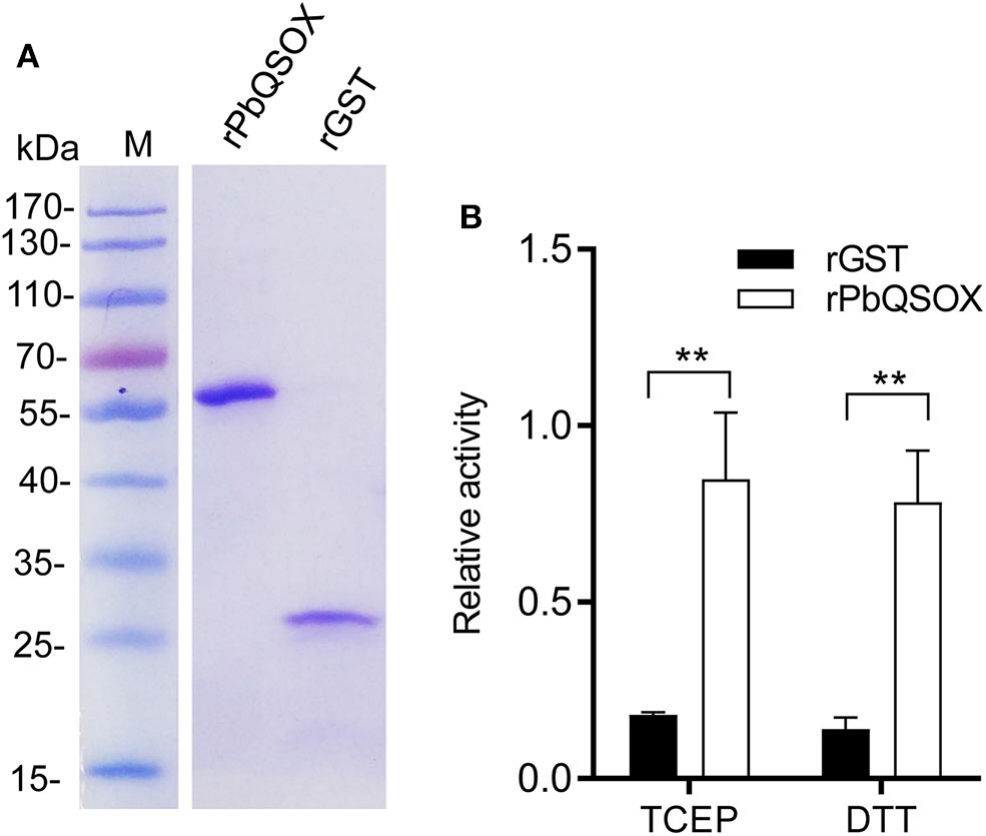
Purification and the oxidase activity of rPbQSOX. **(A)** rPbQSOX and rGST protein were expressed and purified from *E. coli*. Molecular weight of rPbQSOX and the GST-tag protein are ~58 and 26 kDa, respectively. **(B)** The oxidase activities of rPbQSOX with TCEP and DTT as substrates. The oxidase activity was based on determining the rate of the H_2_O_2_ generation. Means were collated from three separate experiments. Recombinant GST (rGST) protein was used as a negative control. Error bars are standard deviations. **p < 0.01.

We wanted to determine whether PbQSOX possessed thiol oxidase activity, given it has the conserved domain structure typical of QSOX proteins. We expressed the rPbQSOX protein lacking the predicted signal peptide in *E. coli*, and purified the protein under native conditions. In addition, we also expressed and purified the rGST protein to serve as the control. SDS-PAGE analysis of rPbQSOX showed a homogeneous band of ~58 kDa, consistent with its predicted molecular size (Figure 2A). We tested rPbQSOX for thiol oxidase activity using the TCEP and DTT as the substrates. QSOX oxidizes TCEP or DTT to produce H_2_O_2_, which can be detected by a luminescent reaction. As shown in Figure 2B, the rPbQSOX possessed obvious oxidase activity, consistent with its orthologs in other eukaryotes (Jaje et al., 2007; Kodali and Thorpe, 2010; Zheng et al., 2012).

### PbQSOX Is Expressed Primarily in Sexual Stages

**Figure 3:**
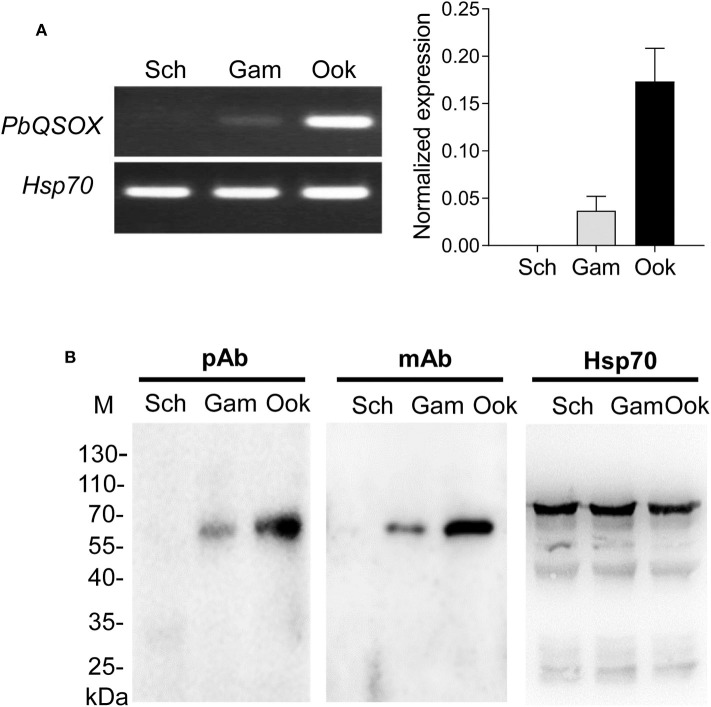
PbQSOX expression during asexual and sexual development. **(A)** *Pbqsox* expression detected by qRT-PCR. Left panel: RT-PCR products of *pbqsox* (upper) and *hsp70* (lower) performed using RNA purified from schizonts (Sch), gametocytes (Gam) and ookinetes (Ook) were separated in 1% agarose gel to show the relative abundance of *pbqsox* transcripts in different stages. Right panel: Quantitation of the *pbqsox* mRNA in different stages by qRT-PCR with the mRNA levels normalized against the house-keeping gene *hsp70*. **(B)** Western blots of PbQSOX in different stages of the parasites. Lysates of schizonts (Sch), gametocytes (Gam), and ookinetes (Ook) at 10 μg/lane were probed with mouse anti-rPbQSOX antisera (pAb, 1:500) and monoclonal antibody (mAb, 1:1,000). Protein loading was estimated by using the antisera against Hsp70 (1:500).

We next investigated the expression of PbQSOX during parasite development. Schizonts, gametocytes and ookinetes were purified on Nycodenz gradients (Figure S2) and used for qRT-PCR and Western blot analysis. *Pbqsox* transcripts were not detected in schizonts, but were detected in gametocytes and ookinetes, with the highest abundance in ookinetes (Figure 3A). To detect the PbQSOX protein expression, mice were immunized with rPbQSOX to produce both polyclonal antisera and a mAb. Western blot analysis of protein lysates from purified schizonts, gametocytes and ookinetes using both anti-PbQSOX mAb and the polyclonal antisera only detected a ~61 kDa protein in gametocytes and ookinetes (Figure 3B). Consistent with the qRT-PCR result, the PbQSOX protein level was much higher in ookinetes than in gametocytes (Figure 3B).

### PbQSOX Is Secreted and Associated With Plasma Membranes of Sexual Stages

**Figure 4:**
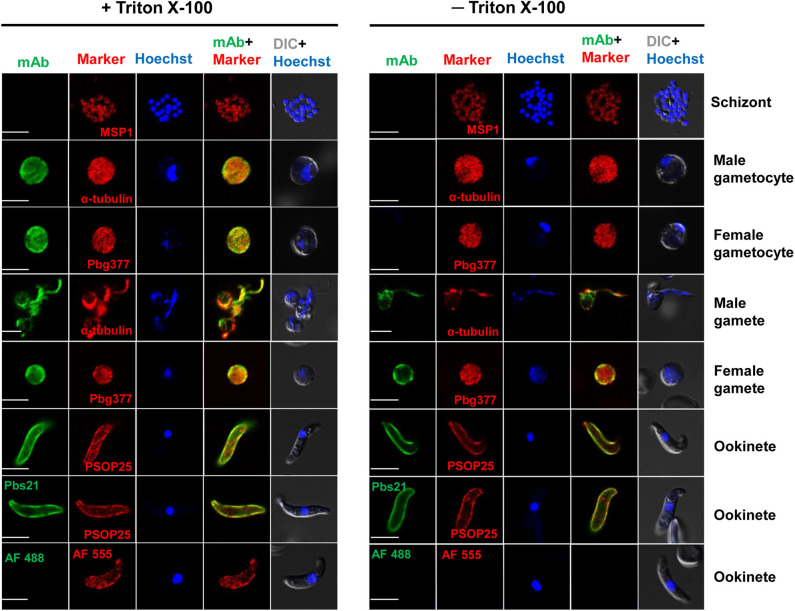
Localization of PbQSOX by IFA. Cells treated with (left panel) or without (right panel) 0.1% Triton X-100 are compared. Anti-rPbQSOX mAb (1:500) was used as the primary antibodies. The antibodies against PbMSP1 for schizonts, Pbg377 for female gametocytes/gametes, α-tubulin for male gametocytes/gametes, and PSOP25 for ookinetes were used as stage-specific markers (red). Nuclei were stained with Hoechst (1:1,000) (blue). Pbs21 mAb was used as positive control for ookinete surface. WT ookinetes labeled only with the secondary antibodies (AF488, Alexa Fluor 488, and AF555, Alexa Fluor 555) were used as a negative control. Two right columns show the merge of PbQSOX + stage-specific marker and merge of nuclear stain (Hoeschst) + DIC (differential interference contrast). Scale bars = 5 μm.

The presence of a putative signal peptide in PbQSOX suggests that it may be a secreted protein. Examination of PbQSOX localization in different stages of *P. berghei* using the anti-PbQSOX mAb detected strong fluorescence in gametocytes, gametes and ookinetes, but not in schizonts or when only the secondary antibodies were used (Figure 4). Furthermore, the fluorescence in gametocytes was observed only after membrane permeabilization, suggesting that PbQSOX was localized in the cytoplasm. In exflagellating microgametes, fluorescence was associated with both the flagella and residual body. In macrogametes and ookinetes, fluorescence was even detected without membrane permeabilization, suggesting PbQSOX was associated with the plasma membrane in these stages. The surface staining of ookinetes with the anti-rPbQSOX mAb was similar to that observed with the anti-Pbs21 mAb (Figure 4). A similar fluorescent pattern was observed using the anti-rPbQSOX polyclonal antisera (Figure S3A). No PbQSOX expression was detected in sporozoites (Figure S3B).

### PbQSOX Is Required for Parasite Sexual Development

To determine the role of PbQSOX during the *Plasmodium* development, a *pbqsox* knockout line, Δ*pbqsox*, was generated using a double cross-over homologous recombination strategy (Janse et al., 2006). Pyrimethamine-resistant parasites were selected and cloned for genotype and phenotype analyses. Deletion of the *pbqsox* gene was confirmed by both integration-specific PCR, and Western blot (Figure S4).

**Figure 5:**
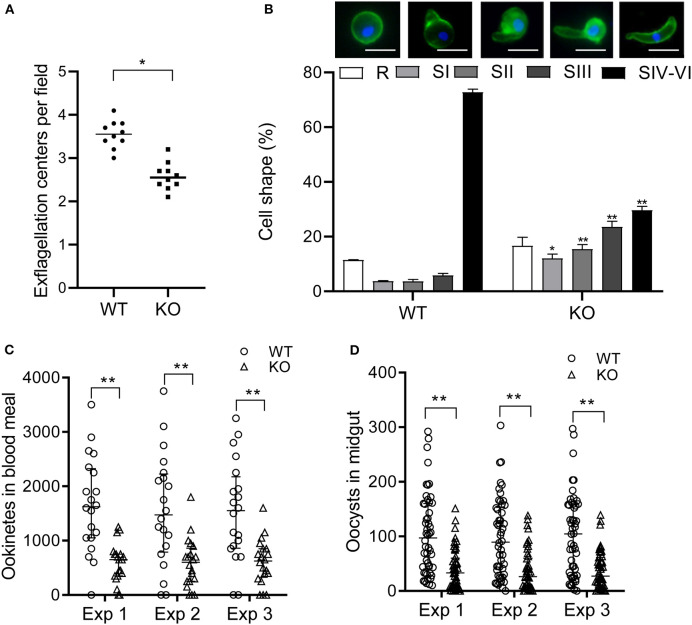
Phenotypic analyses of the Δ*pbqsox* parasites. **(A)** Exflagellation of WT or Δ*pbqsox* (KO) microgametocytes. Exflagellation centers were counted under a phase contrast microscope at 400× magnification. Individual data points represent the number of exflagellation centers from each mouse. Horizontal bars indicate the median number of exflagellation centers. **p < 0.01. **(B)** Ookinete stages in *in vitro* cultures of WT and Δ*pbqsox* (KO) parasites. Morphologies of ookinetes used for defining different stages are given above the graph. Scale bars = 5 μm. Means were obtained from three separate experiments. Error bars indicate standard error of the mean (SEM) within individual treatments. * and ** *p* < 0.05 and <0.01, respectively, for comparison of the respective cell shapes between WT and KO. **(C)** Ookinete number in the blood meal per mosquito after feeding on WT- and Δ*pbqsox* (KO)*-*infected mice. Three independent experiments were performed. **p < 0.01. Horizontal bars indicate the median with interquartile range of individual treatments. **(D)** Oocyst number per midgut in mosquitoes 10 days after blood feeding on WT- or Δ*pbqsox* (KO)-infected mice. Three independent experiments were performed. **p < 0.01. Horizontal bars indicate the median with interquartile range of individual treatments.

To determine the effect of *pbqsox* deletion on parasite development, equal numbers of the WT- and Δ*pbqsox*-iRBCs were injected i.p. into BALB/c mice, and parasitemia was determined daily in Giemsa-stained thin blood smears, while gametocytemia and gametocyte sex ratio were determined on day 3 p.i. Consistent with the lack of PbQSOX expression in asexual erythrocytic stages, *pbqsox* deletion had no noticeable effect on asexual parasitemia (Figure S5A). In addition, it did not affect the gametocytogenesis, as gametocytemia (Figure S5B) and gametocyte sex ratio (Figure S5C) did not differ significantly between the WT and the Δ*pbqsox* parasites. Whereas, *pbqsox* deletion did not affect the formation of macrogametes (Figure S5D), it reduced the number of exflagellation centers by 28.0% (*p* < 0.05; Figure 5A). Subsequently, *in vitro* ookinete culture showed that ookinetes in the Δ*pbqsox* line retained normal morphology (Figure S6A), but the proportion of ookinetes (stage IV-VI) was reduced by 43.1% (*p* < 0.01; Figure 5B). Detailed analysis of ookinete differentiation showed that 12%, 15.4%, and 23.5% of the Δ*pbqsox* ookinetes arrested at stage I, II, and III, respectively, as compared to 3.7%, 3.6% and 5.8% at these stages for the wild-type control (*p* < 0.01; Figure 5B), suggesting that *pbqsox* deletion interfered with the zygote–ookinete maturation process.

The effect of *pbqsox* deletion on parasite development was further evaluated in mosquito feeding assays. *Anopheles stephensi* mosquitoes were allowed to feed on Δ*pbqsox*- and WT parasite-infected mice and the formation of ookinetes and oocysts were evaluated 24 h and 10 days post blood feeding, respectively. Consistent with the *in vitro* ookinete conversion result, the number of ookinetes formed in the blood bolus of mosquitoes feeding on Δ*pbqsox*-infected mice was 60.2–64.6% lower than that with the WT parasites (*p* < 0.01; Figure 5C, Table 1). Although there was no reduction in the prevalence of infected mosquitoes between Δ*pbqsox* and WT parasites (Table 1), there was a 61.8–62.8% reduction in oocyst number/midgut in mosquitoes after feeding on Δ*pbqsox*-infected mice as compared to those fed on WT parasite-infected mice (*p* < 0.01; Figure 5D). Of note, the gross morphology of oocysts appeared normal in the Δ*pbqsox* parasites compared to the WT (Figure S6B). Together, these findings indicate that PbQSOX plays an important role in male gametogenesis, and ookinete maturation.

**Table 1:** The effect of *pbqsox* deletion on ookinete and oocyst development in mosquitoes.

Exp^*^	**Group**	**Ookinetes in blood meal at 24 h post feeding**				**Oocysts in midgut at 10 days post feeding**					
		**# Infected/Dissected (%)**	**# ookinetes/midgut [mean (range)]**	**% reduction of ookinetes^a^**	***P*^b^**	**# Infected/Dissected (%)**	**% reduction of prevalence^c^**	***P*^d^**	**# oocysts/midgut[mean (range)]**	**% reduction of oocyst density^e^**	***P*^f^**
1	WT	19/20 (95)	1,663 (0–3,500)	64.6	0.000	50/50 (100)	6	0.241	103.9 (10–292)	62.8	0.000
	*Δpbqsox*	18/20 (90)	587.5 (0–1,250)			47/50 (94)			38.6 (0–151)		
2	WT	18/20 (90)	1,560 (0–3750)	62.5	0.001	49/50 (98)	12	0.065	97.2 (0–303)	61.8	0.000
	*Δpbqsox*	17/20 (85)	585 (0–1,800)			43/50 (86)			37.1 (0–138)		
3	WT	18/20 (90)	1,533 (0–3,250)	60.2	0.000	49/50 (98)	6	0.359	102.8 (0–297)	62.3	0.000
	*Δpbqsox*	17/20 (85)	610 (0–1,600)			46/50 (92)			38.6 (0–139)		

* *The study was performed in three experiments (Exp), and the numbers of ookinetes and oocysts in each midgut were determined at 24 h and 10 days post blood feeding, respectively*.

a *% reduction in ookinetes was calculated as (mean _WT_–mean _Δpbqsox_)/mean _WT_ × 100%*.

b *P values from Mann–Whitney U test for comparison between the WT and Δpbqsox groups*.

c *% reduction in prevalence was calculated as % prevalence _WT_–% prevalence _Δpbqsox_*.

d *P-values from Fisher's exact test for comparison between the WT and Δpbqsox groups*.

e *% reduction in oocysts was calculated as (mean _WT_–mean _Δpbqsox_)/mean _WT_ × 100%*.

f *P -values from Mann–Whitney U test for comparison between the WT and Δpbqsox groups*.

### PbQSOX Is Required for Disulfide Bridges of Surface Proteins in Ookinetes

**Figure 6:**
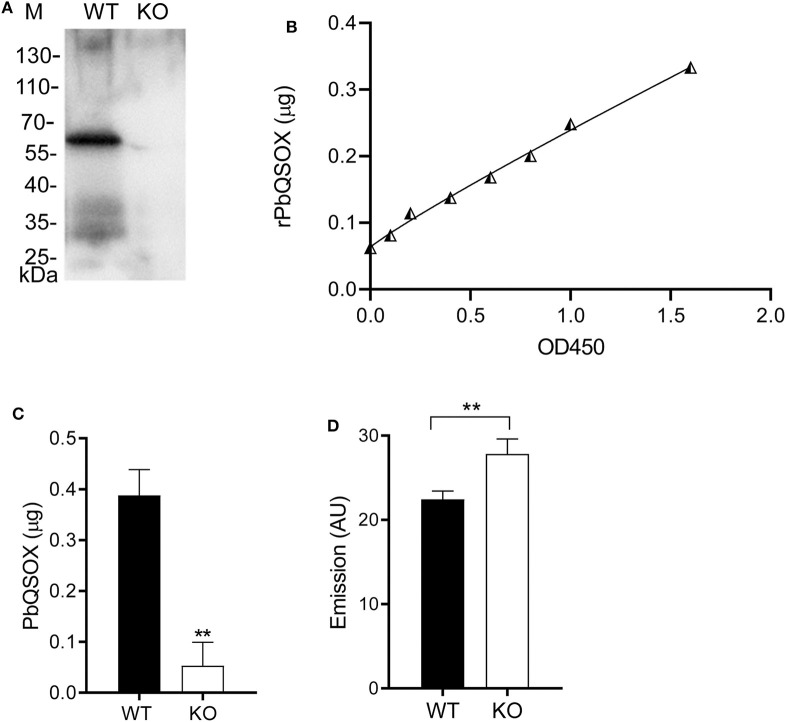
PbQSOX secretion by cultured ookinetes and its potential effect on ookinete surface proteins. **(A)** Western blot analysis of culture supernatants of WT and Δ*pbqsox* (KO) ookinetes probed with anti-rPbQSOX mAb showing the presence of PbQSOX in culture medium. **(B)** Non-linear regression standard curve between rPbQSOX concentrations and optical density readings at OD450 by ELISA. The rPbQSOX at different dilutions was used to coat the wells of the ELISA plate and the standard curve was established to quantify PbQSOX in the ookinete culture medium. **(C)** Quantification of PbQSOX in culture medium. Culture supernatants of 1 × 10^4^ WT and Δ*pbqsox* (KO) ookinetes were used to quantify the PbQSOX by ELISA. **p < 0.01. Values represent the mean and SEM of three independent experiments. **(D)** Reactive thiol content on the WT and Δ*pbqsox* (KO) ookinetes. One hundred ookinetes from each group were labeled with ThioGlo. Emission (AU, arbitrary units) was measured using an ELISA plate reader (excitation at 400 nm and emission at 465 nm). Data indicate the mean and SEM from three independent experiments.

With the presence of a signal sequence and its association with the surface of ookinetes, we reasoned that PbQSOX might be secreted/shed into the medium. To detect secretion/shedding of PbQSOX, equal numbers (1 × 10^4^) of WT and Δ*pbqsox* ookinetes were cultured in the ookinete culture medium for 24 h. Western blot analysis of concentrated culture medium detected PbQSOX in the culture of WT ookinetes, but not in the Δ*pbqsox* ookinetes (Figure 6A). Using the standard curve established with the rPbQSOX ELISA (Figure 6B), we estimated that the amount of PbQSOX secreted or shed by 1 × 10^4^ WT ookinetes into the culture medium to be 0.38 ± 0.05 μg (Figure 6C).

We hypothesized that PbQSOX might be needed to maintain the integrity of disulfide bridges of surface proteins during sexual development, which is required for ookinete development and maturation. To evaluate this possible function, we quantified the surface thiol contents of the WT and Δ*pbqsox* ookinetes using ThioGlo™. The results showed that the thiol content was significantly higher on the Δ*pbqsox* ookinetes than that on the WT ookinetes (*p* < 0.01; Figure 6D), indicating the presence of more reduced proteins on the mutant ookinetes.

### Antibodies Against PbQSOX Showed Obvious TB Activities

The surface association of PbQSOX with sexual stages prompted us to test whether antibodies against PbQSOX possess TB activities. Immunization of BALB/c mice with purified rPbQSOX elicited an effective antibody response. Antibody titers increased over time following the initial immunization, with substantial boosting observed in the two subsequent immunizations (data not shown). The anti-rPbQSOX mAb produced from a selected hybridoma line was purified, and the isotype was determined to be IgG2b. Both anti-rPbQSOX sera and mAb were then used in *in vitro* ookinete conversion and mosquito feeding assays.

**Figure 7:**
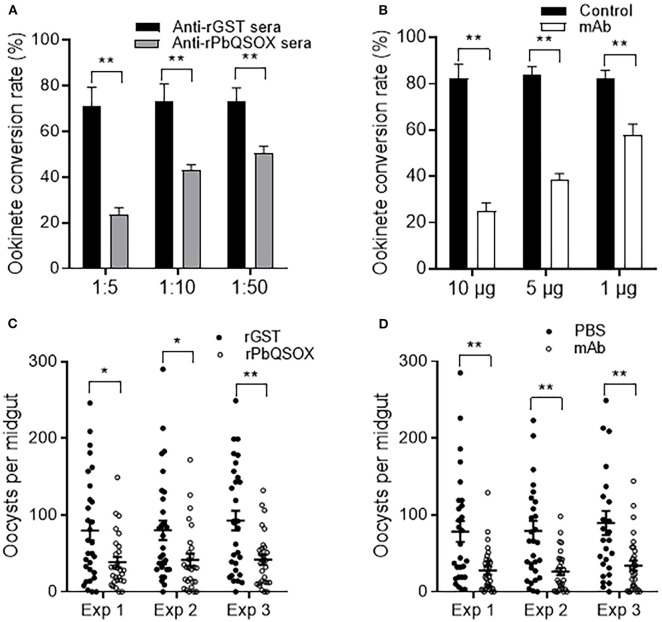
Transmission-blocking activities of anti-rPbQSOX antibodies. **(A)** Effect of anti-rPbQSOX antisera on *P. berghei* ookinete formation *in vitro*. Anti-rGST sera were used as control. Sera were used at final dilutions of 1:5, 1:10, and 1:50. **(B)** Effect of anti-rPbQSOX mAb on *P. berghei* ookinete formation *in vitro*. Anti-rPbQSOX mAb was added at 10, 5 and 1 μg/100 μl in cultures. Cultures without mAb were used as a negative control. Means were collated from three separate experiments. Data in **A** and **B** indicate mean and SEM from three separate experiments. **p < 0.01. **(C)** Direct mosquito feeding assay on mice immunized with rPbQSOX and rGST control. **(D)** Passive antibody transfer experiment to assess the TB activity of the anti-rPbQSOX mAb and PBS control. Data in **(C,D)** were from three independent experiments (Exp 1 – 3). Individual data points represent the number of oocysts found in individual mosquitoes 10 days post-feeding. Horizontal bars indicate the median with interquartile range of individual experiments. *p < 0.05, **p < 0.01.

Ookinete conversion was determined by culturing parasites for 24 h in an ookinete culture medium containing mouse anti-rPbQSOX sera or mAb. In both cases, the antisera and the mAb inhibited ookinete conversion in a dose-dependent manner. In ookinete cultures supplemented with the anti-rPbQSOX sera at 1:5, 1:10 and 1:50 dilutions, ookinete conversion rates were reduced by 66.9, 41.3, and 30.8%, respectively (*p* < 0.01), as compared with the anti-rGST control sera (Figure 7A). In cultures with the anti-rPbQSOX mAb added at 10, 5 and 1 μg/100 μl, ookinete conversion rates were reduced by 69.7, 54.1, and 29.3%, respectively (*p* < 0.01; Figure 7B).

To examine the TB effect of anti-rPbQSOX antibodies *in vivo*, mice were immunized with rPbQSOX or the control rGST protein. Ten days after the last immunization, six immunized mice in each group were infected with the WT *P. berghei* to assess TB activity by the direct feeding assay. On day 10 post feeding, mosquitoes were dissected and midgut oocysts were counted. Whereas the prevalence of infected mosquitoes was similar after feeding on rGST- and rPbQSOX-immunized mice (Table 2), oocyst density in mosquitoes that fed on rPbQSOX-immunized mice was reduced by 51.7, 48.1, and 55%, respectively, as compared to the rGST-immunized control mice (Figure 7C, Table 2). Next, we evaluated the TB effect of the anti-rPbQSOX mAb in an antibody transfer experiment, where mice infected with WT *P. berghei* were injected intravenously with either 150 μg of anti-rPbQSOX mAb/mouse or PBS 1 h before mosquito feeding. Compared with mosquitoes that fed on the control mice, those fed on mAb-transferred mice showed reductions in oocyst density by 64.6, 66.6, and 62.0%, respectively (Figure 7D, Table 2), although the prevalence of infected mosquitoes was not different between the two groups.

**Table 2:** *In vivo* evaluation of transmission–blocking effect of anti-rPbQSOX sera and monoclonal antibody (mAb).

**Exp**	**Immunization groups**							**mAb transfer groups**						
	**Immunization**	**# Infected/dissected (%)**	**% RP^a^**	***P*^b^**	**# oocyst/midgut [mean (range)]**	**% ROD^c^**	***P*^d^**	**Treatment**	**# Infected/dissected (%)**	**% RP^a^**	***P^b^***	**# oocyst/midgut [mean (range)]**	**% ROD^c^**	***P^d^***
1	rGST	28/30 (93.3)	0.5	1.000	79.8 (0–246)	51.7	0.021	PBS	28/28 (100)	10	0.261	78.4 (3–285)	64.6	0.002
	rPbQSOX	26/28 (92.8)			38.5 (0–149)			mAb	27/30 (90)			27.7 (0–129)		
2	rGST	29/30 (96.6)	7.8	0.530	80.3 (0–290)	48.1	0.013	PBS	29/30 (96.6)	10.4	0.330	79.1 (0–301)	66.6	0.001
	rPbQSOX	24/27 (88.8)			41.6 (0–172)			mAb	25/29 (86.2)			26.4 (0–98)		
3	rGST	29/30 (96.6)	3.8	0.951	92.9 (0–249)	55	0.003	PBS	25/26 (96.1)	9.5	0.440	89.5 (0–305)	62.0	0.002
	rPbQSOX	26/28 (92.8)			41.8 (0–132)			mAb	26/30 (86.6)			34.0 (0–144)		

a *RP: % reduction in prevalence was calculated as % prevalence _rGST or PBS_ – % prevalence _rPbQSOXormAb_*.

b *P-values from Fisher's exact test for comparison between the control rGST or PBS and rPbQSOX immunization or mAb transfer groups*.

c *ROD: % reduction in oocyst intensity was calculated as (mean _rGST or PBS_ – mean _rPbQSOX or mAb_)/mean _rGST or PBS_ × 100%*.

d *P-values from Mann–Whitney U test for comparison between control rGST or PBS and rPbQSOX immunization or mAb transfer groups*.

## Discussion

During its development in the erythrocytes of the vertebrate host, the malaria parasite endures a substantial amount of intracellular oxidative stress resulting from hemoglobin catabolism (Muller, 2004), while inside the blood bolus after ingestion by a mosquito, it is exposed to extracellular oxidative insults from the digestive enzymes of the mosquito and immune factors of the vertebrate host (Sinden et al., 1996; Margos et al., 2001). The malaria parasite possesses two major antioxidant systems, the glutathione system and the thioredoxin system, to deal with these oxidative stresses (Muller, 2004). These antioxidant systems are not only important for the asexual development, but also are induced during ookinete development in the mosquito midgut (Turturice et al., 2013). Here, we identified QSOX as another antioxidant system involved in the regulation of the redox state and possibly folding of proteins in malaria parasites. QSOX is a specialized enzyme that catalyzes the introduction of disulfide bonds into unfolded reduced proteins (Thorpe et al., 2002; Thorpe and Coppock, 2007; Heckler et al., 2008). QSOX is conserved in *Plasmodium* and also other protozoan parasites such as trypanosomes, and the PbQSOX protein possesses thiol oxidase activity similar to vertebrate QSOX proteins (Kodali and Thorpe, 2010), despite the fact that protozoan QSOXs contain only a single thioredoxin domain.

*Plasmodium* QSOX appears to play an important role in sexual development of the parasite, since deletion of *pbqsox* resulted in defects in sexual development, leading to remarkable reductions in the numbers of mature ookinetes and oocysts. PbQSOX is expressed primarily during sexual development and is associated with the surface of gametes and ookinetes. In addition, *P. berghei* ookinetes also secrete/shed large amounts of PbQSOX into the medium during *in vitro* culture. Analogously, QSOX enzymes have been found in secreted fluids including milk, semen, egg white and blood serum (Hoober et al., 1996; Benayoun et al., 2001; Zanata et al., 2005; Jaje et al., 2007). The extracellular QSOXs in different biological systems suggest they may play diverse roles (Limor-Waisberg et al., 2013). For instance, it was found that extracellular catalysis of disulfide bond formation by human QSOX1 is needed for laminin incorporation into the extracellular matrix, which is a prerequisite for tumor adhesion and metastasis (Ilani et al., 2013). In this context, we found that *pbqsox* deletion resulted in reduced numbers of mature ookinetes with concomitant accumulation of earlier stages, implying that PbQSOX is needed during ookinete development. The identification of significantly increased thiol groups on Δ*pbqsox* ookinetes as compared to those on the WT ookinetes indicates that PbQSOX plays a critical role in maintaining the structural integrity of ookinete surface proteins during the extracellular development of ookinetes. Further, whether PbQSOX is expressed and plays a role during liver stage development warrants future studies.

The abundant expression and critical function of PbQSOX during sexual development provide a potential target for blocking parasite transmission to mosquitoes. The expression of PbQSOX on both pre-fertilization and post-fertilization stages imply that antibodies against this protein may interrupt parasite transmission at multiple steps, e.g., preventing mating and formation of zygotes (Wu et al., 2015; Bechtsi and Waters, 2017) and subsequent transition and maturation of ookinetes (Guttery et al., 2015). We observed that anti-rPbQSOX antibodies significantly inhibited ookinete formation *in vitro*. The levels of inhibition at higher antiserum concentrations (1:5 dilution) compared favorably with that for the anti-*P. berghei* HAP2 serum, which inhibited ookinete formation up to ~81% (Blagborough and Sinden, 2009). Moreover, we also observed PbQSOX on the surface of ookinetes, suggesting that anti-PbQSOX antibodies may also interfere with the invasion of the midgut epithelium and formation of oocysts (Smith and Barillas-Mury, 2016). In direct feeding experiments, oocyst density in mosquitoes that fed on rPbQSOX-immunized mice was also moderately reduced, which was similar to the TB efficiency of the ookinete secreted protein PSOP12 (Sala et al., 2015). Altogether, these results demonstrated evident TB activity of PbQSOX antibodies, which warrants further studies on the human malaria parasites.

In summary, we find that PbQSOX is a conserved *Plasmodium* protein and is required for parasite sexual development especially for ookinete maturation. PbQSOX is expressed primarily in both pre- and post-fertilization stages, and antibodies against PbQSOX showed obvious TB activities at ookinete steps, highlighting the TBV potential of PbQSOX.

## Data Availability Statement

The QSOX protein sequences were downloaded from the GenBank (https://www.ncbi.nlm.nih.gov/genbank/) with accession numbers NP_508419.1 (*Caenorhabditis elegans*), NP_001121836.1 (*Danio rerio*), NP_705787.1 (*Mus musculus*), NP_002817.2 (*Homo sapiens*), NP_565258.1 (*Arabidopsis thaliana*), and XP_011773962.1 (*Trypanosoma brucei*).

## Ethics Statement

The animal study was reviewed and approved by The Animal Usage Committee of China Medical University.

## Author Contributions

LC and YC conceived the study. WZ and FL designed and performed experiments and data analysis, and drafted the paper. FD, XK, FY, YH, HF, and HM performed experiments and data analysis. QF, EL, and JM participated in data analysis and edited the paper. All authors contributed to the article and approved the submitted version.

## Conflict of Interest

The authors declare that the research was conducted in the absence of any commercial or financial relationships that could be construed as a potential conflict of interest.
