# Supplementary material for: Characterization of a Sulfhydryl Oxidase From Plasmodium berghei as a Target for Blocking Parasite Transmission
Source: Front Cell Infect Microbiol. 2020 Jun 26;10:311. doi: 10.3389/fcimb.2020.00311 (PMC7332561; doi:10.3389/fcimb.2020.00311)
Supplement: Supplementary file 1 [file Table_1.docx]

**Table S1. Primer information and sequences.**

| Purpose | Primer name | Sequence |
| --- | --- | --- |
| RT-PCR | Pbqsox1 | AAAAGGGTGAAGTAGTGT |
|  | Pbqsox2 *Pbqsox* | ATGCTGGGCAATAATA |
|  | Hsp70F | GTATTATTAATGAACCCACCGCT |
|  | Hsp70R | GAAACATCAAATGTACCACCTCC |
| Expression of rPbQSOX | *pbqsox*-F | CGCGGATCCGACGACGACGACAAGTCGGAACATTCAGATATTTGCA |
|  | *pbqsox*-R | CCCAAGCTTCTATGAATATATGCTGACC |
| PbQSOX KO | 5UTR-F | CCCAAGCTTTTGTTTGGTCTCGCTG |
|  | 5UTR-R | AAAACTGCAGCGTTGATTTAGCTTTTTCG |
|  | 3UTR-F | CCGCTCGAGTTTATCCTACGATTTCACTTTTTT |
|  | 3UTR-R | CCGGAATTCAATACAATGACGCTTCTAACGA |
| Δ*pbqsox* line integration-specific PCR | Primer 1 | TATGAAAACGCTAATACTAGTCCA |
|  | Primer 2 | TGAATGTTCCGAACCCCA |
|  | Primer 3 | GGTGCTTTGAGGGGTGAG |
