# Supplementary material for: Characterization of a Sulfhydryl Oxidase From Plasmodium berghei as a Target for Blocking Parasite Transmission
Source: Front Cell Infect Microbiol. 2020 Jun 26;10:311. doi: 10.3389/fcimb.2020.00311 (PMC7332561; doi:10.3389/fcimb.2020.00311)
Supplement: Supplementary file 2 [file Presentation_1.PPTX]

## Slide 1
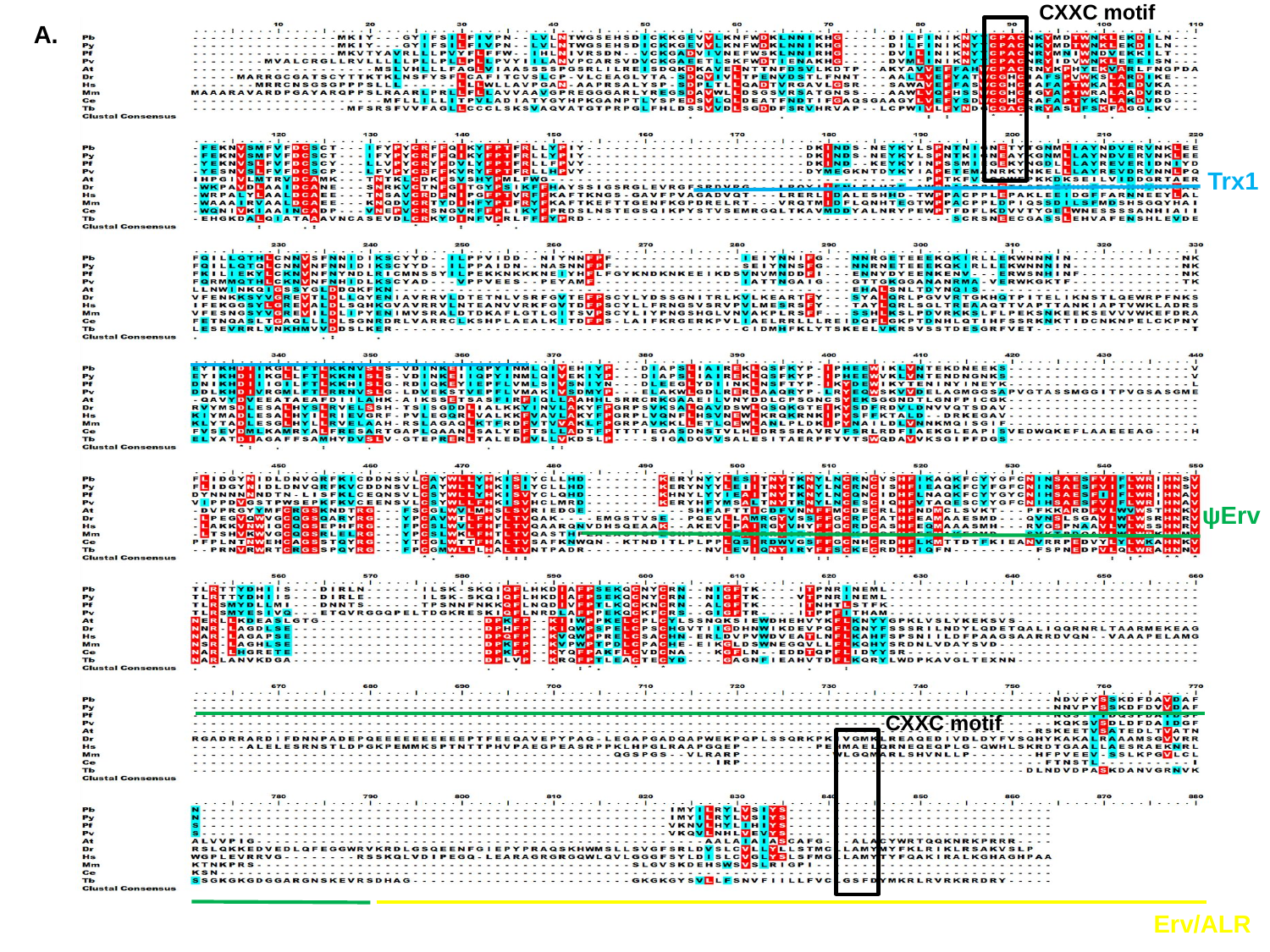

CXXC motif
A.
Trx1
ψErv
CXXC motif
Erv/ALR

## Slide 2
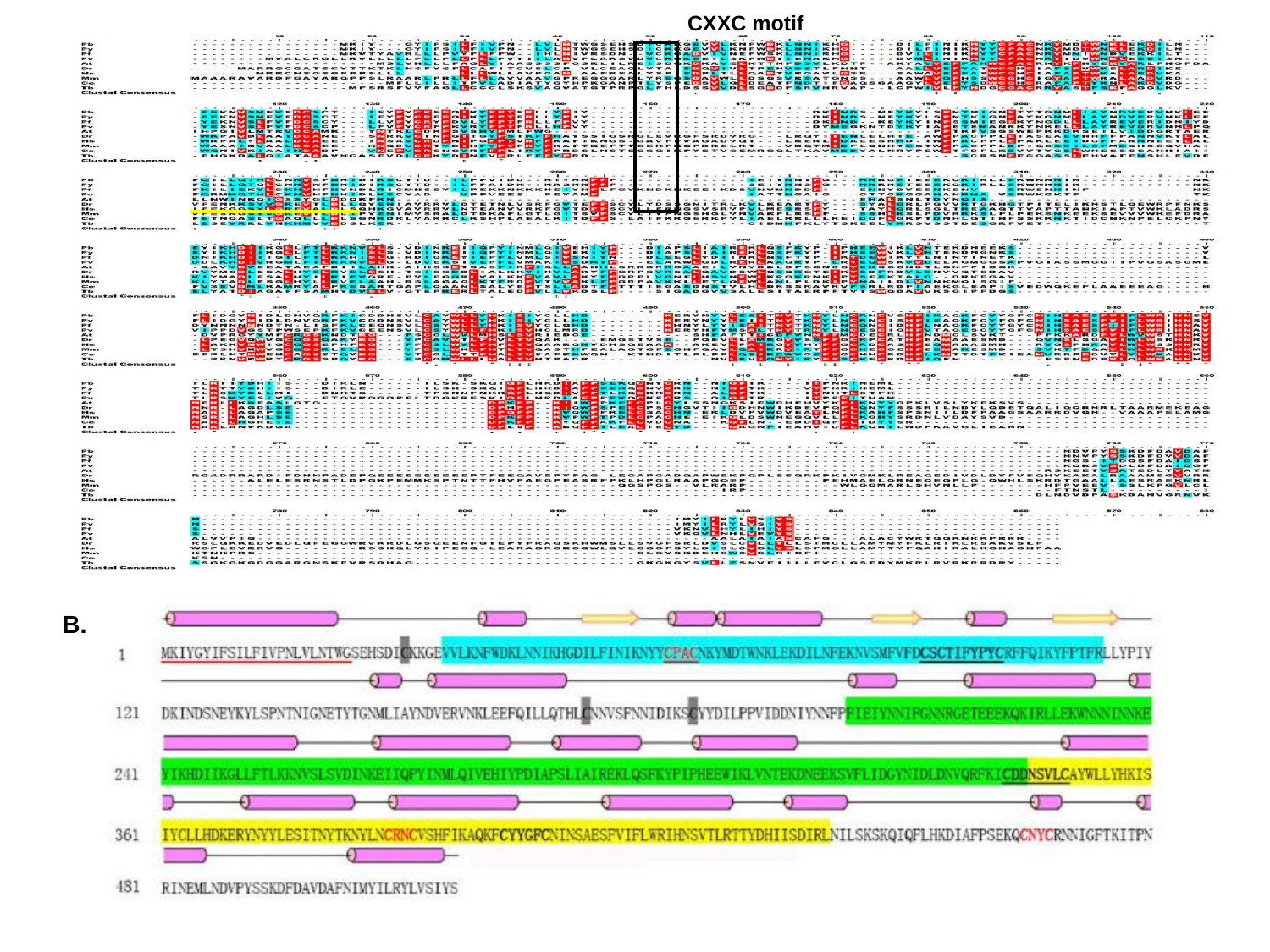

CXXC motif
B.

## Slide 3
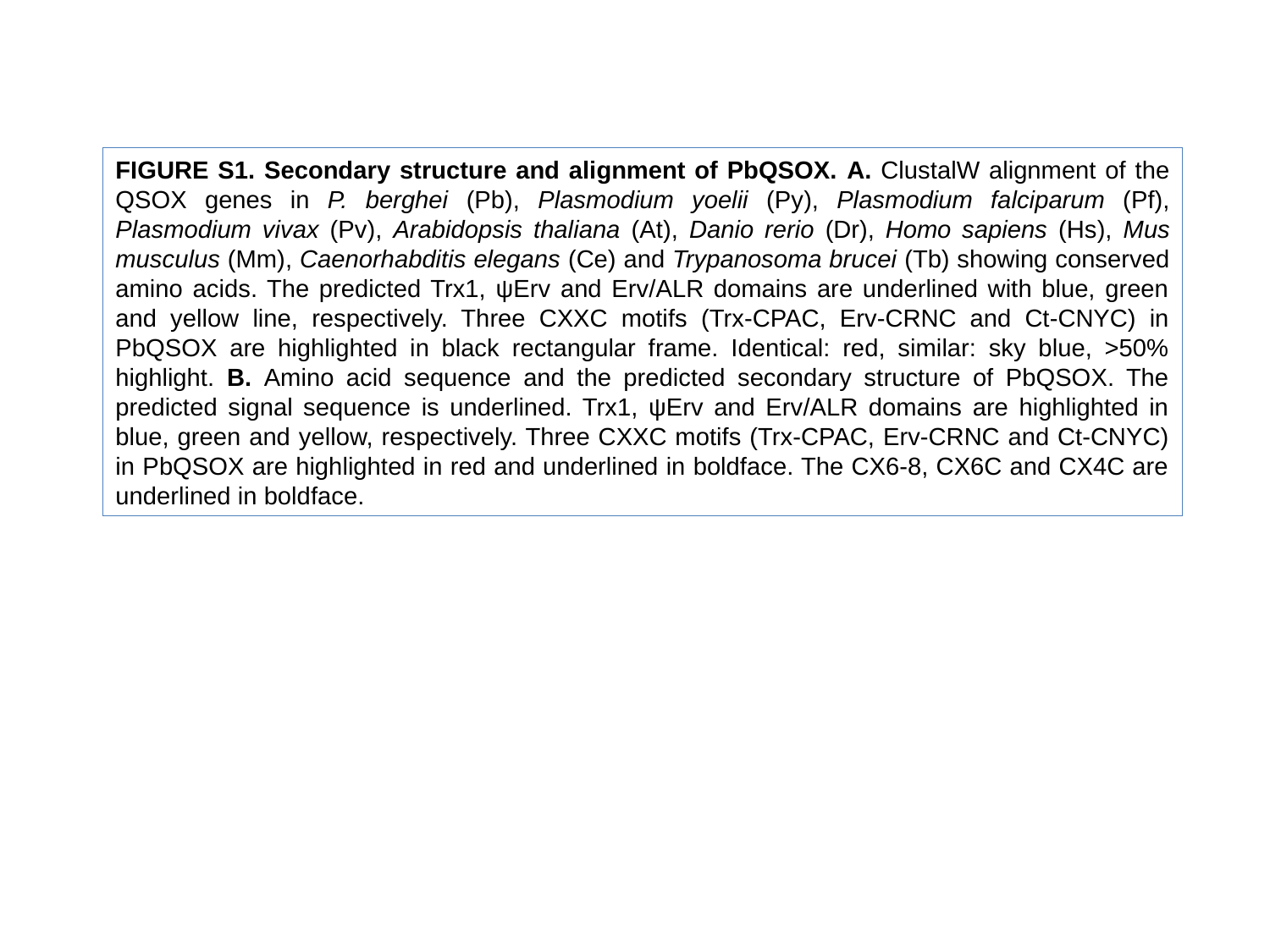

FIGURE S1. Secondary structure and alignment of PbQSOX. A. ClustalW alignment of the QSOX genes in P. berghei (Pb), Plasmodium yoelii (Py), Plasmodium falciparum (Pf), Plasmodium vivax (Pv), Arabidopsis thaliana (At), Danio rerio (Dr), Homo sapiens (Hs), Mus musculus (Mm), Caenorhabditis elegans (Ce) and Trypanosoma brucei (Tb) showing conserved amino acids. The predicted Trx1, ψErv and Erv/ALR domains are underlined with blue, green and yellow line, respectively. Three CXXC motifs (Trx-CPAC, Erv-CRNC and Ct-CNYC) in PbQSOX are highlighted in black rectangular frame. Identical: red, similar: sky blue, >50% highlight. B. Amino acid sequence and the predicted secondary structure of PbQSOX. The predicted signal sequence is underlined. Trx1, ψErv and Erv/ALR domains are highlighted in blue, green and yellow, respectively. Three CXXC motifs (Trx-CPAC, Erv-CRNC and Ct-CNYC) in PbQSOX are highlighted in red and underlined in boldface. The CX6-8, CX6C and CX4C are underlined in boldface.

## Slide 4
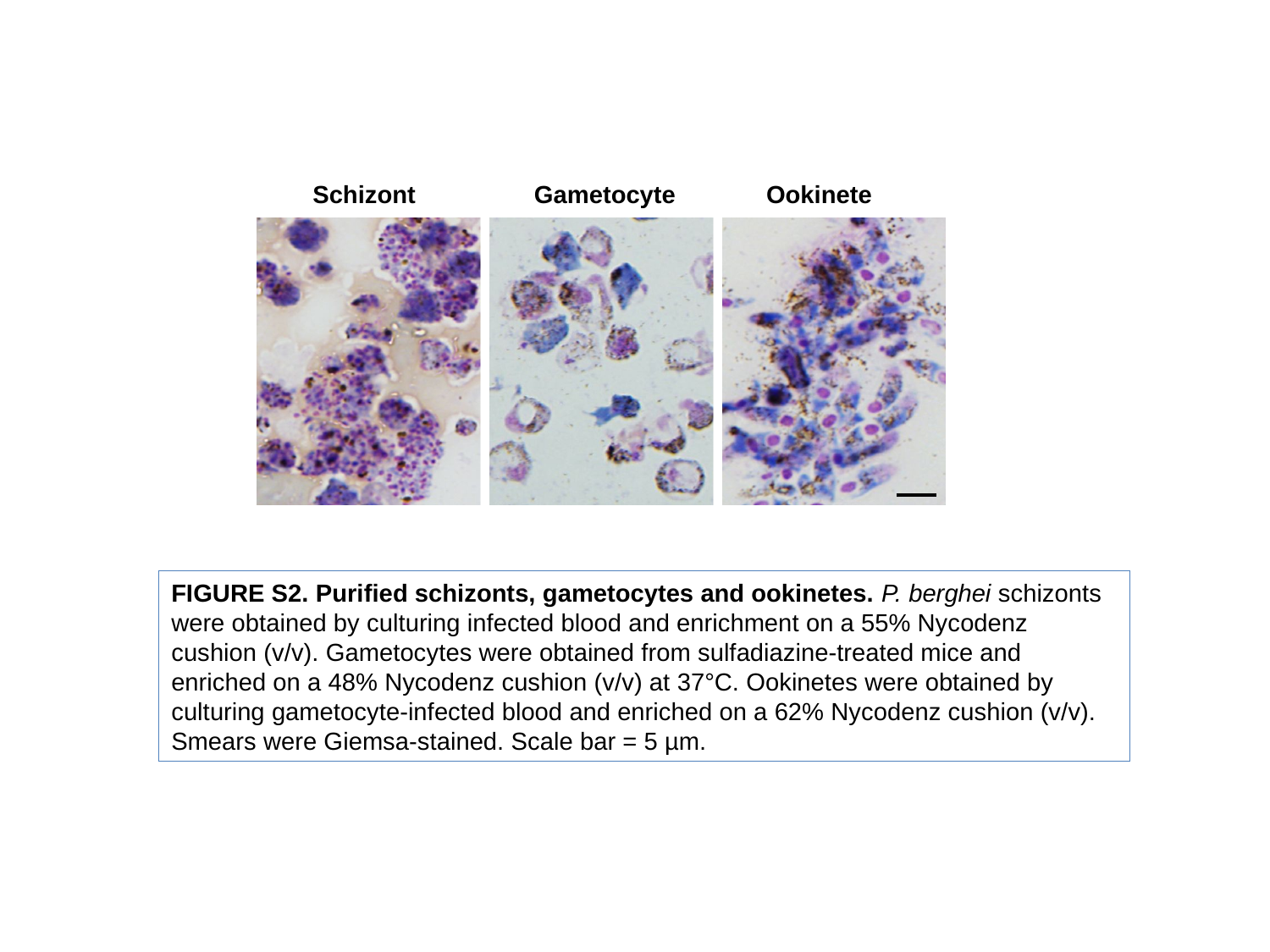

Schizont Gametocyte Ookinete
FIGURE S2. Purified schizonts, gametocytes and ookinetes. P. berghei schizonts were obtained by culturing infected blood and enrichment on a 55% Nycodenz cushion (v/v). Gametocytes were obtained from sulfadiazine-treated mice and enriched on a 48% Nycodenz cushion (v/v) at 37°C. Ookinetes were obtained by culturing gametocyte-infected blood and enriched on a 62% Nycodenz cushion (v/v). Smears were Giemsa-stained. Scale bar = 5 µm.

## Slide 5
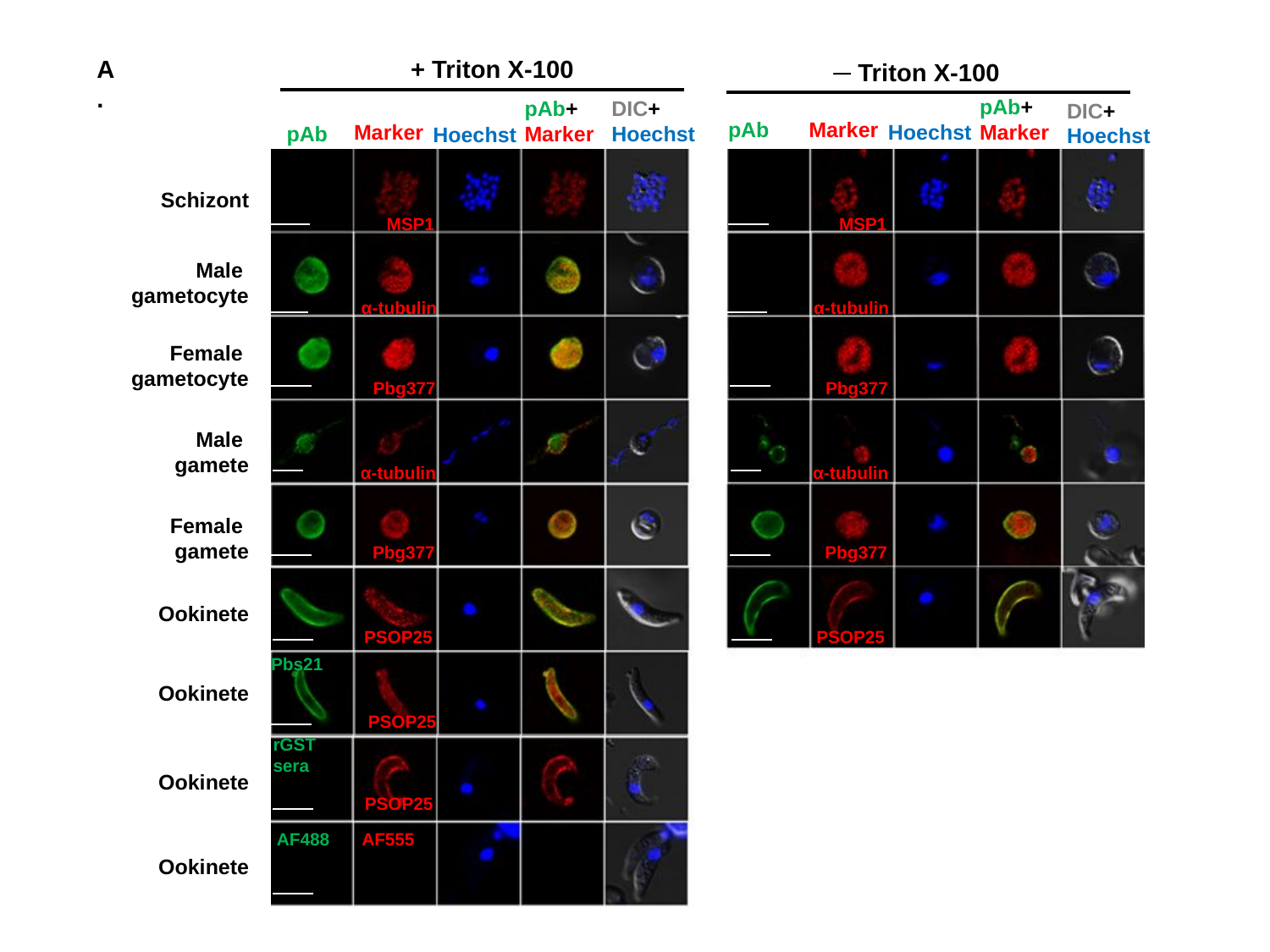

A.
+ Triton X-100
─ Triton X-100
pAb+
Marker
pAb+
Marker
DIC+
Hoechst
DIC+
Hoechst
pAb
Marker
Hoechst
Marker
pAb
Hoechst
Schizont
MSP1
MSP1
Male
gametocyte
α-tubulin
α-tubulin
Female
gametocyte
Pbg377
Pbg377
Male
gamete
α-tubulin
α-tubulin
Female
gamete
Pbg377
Pbg377
Ookinete
PSOP25
PSOP25
Pbs21
Ookinete
PSOP25
rGST
sera
Ookinete
PSOP25
AF488
AF555
Ookinete

## Slide 6
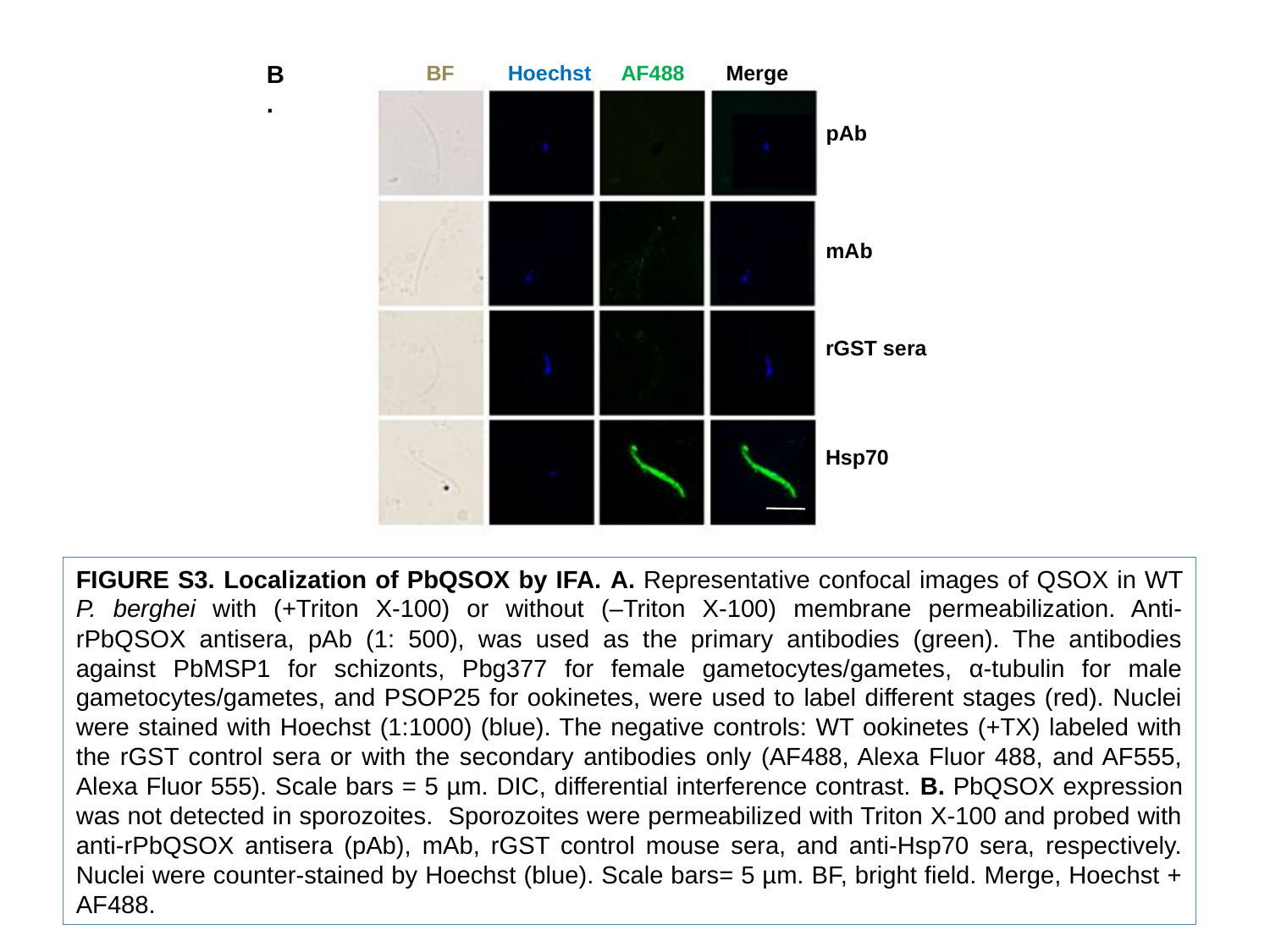

B.
BF Hoechst AF488 Merge
pAb
mAb
rGST sera
Hsp70
FIGURE S3. Localization of PbQSOX by IFA. A. Representative confocal images of QSOX in WT P. berghei with (+Triton X-100) or without (–Triton X-100) membrane permeabilization. Anti-rPbQSOX antisera, pAb (1: 500), was used as the primary antibodies (green). The antibodies against PbMSP1 for schizonts, Pbg377 for female gametocytes/gametes, α-tubulin for male gametocytes/gametes, and PSOP25 for ookinetes, were used to label different stages (red). Nuclei were stained with Hoechst (1:1000) (blue). The negative controls: WT ookinetes (+TX) labeled with the rGST control sera or with the secondary antibodies only (AF488, Alexa Fluor 488, and AF555, Alexa Fluor 555). Scale bars = 5 µm. DIC, differential interference contrast. B. PbQSOX expression was not detected in sporozoites. Sporozoites were permeabilized with Triton X-100 and probed with anti-rPbQSOX antisera (pAb), mAb, rGST control mouse sera, and anti-Hsp70 sera, respectively. Nuclei were counter-stained by Hoechst (blue). Scale bars= 5 µm. BF, bright field. Merge, Hoechst + AF488.

## Slide 7
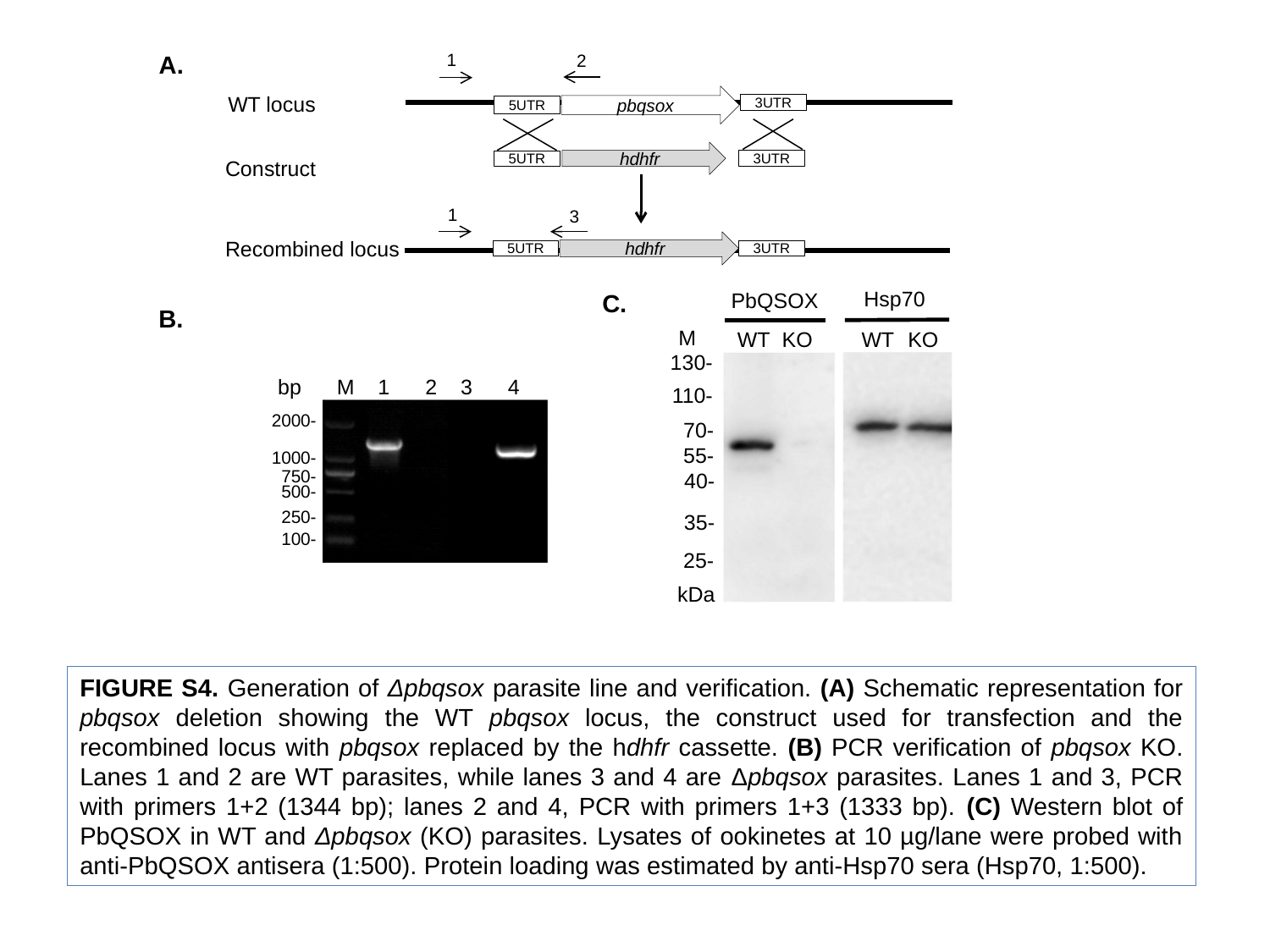

1
2
WT locus
pbqsox
3UTR
5UTR
hdhfr
Construct
3UTR
5UTR
1
3
Recombined locus
hdhfr
5UTR
3UTR
A.
Hsp70
PbQSOX
M
WT
KO
WT
130-
110-
70-
55-
 40-
 35-
 25-
kDa
KO
C.
B.
bp M 1 2 3 4
2000-
1000-
 750-
 500-
 250-
 100-
FIGURE S4. Generation of Δpbqsox parasite line and verification. (A) Schematic representation for pbqsox deletion showing the WT pbqsox locus, the construct used for transfection and the recombined locus with pbqsox replaced by the hdhfr cassette. (B) PCR verification of pbqsox KO. Lanes 1 and 2 are WT parasites, while lanes 3 and 4 are Δpbqsox parasites. Lanes 1 and 3, PCR with primers 1+2 (1344 bp); lanes 2 and 4, PCR with primers 1+3 (1333 bp). (C) Western blot of PbQSOX in WT and Δpbqsox (KO) parasites. Lysates of ookinetes at 10 µg/lane were probed with anti-PbQSOX antisera (1:500). Protein loading was estimated by anti-Hsp70 sera (Hsp70, 1:500).

## Slide 8
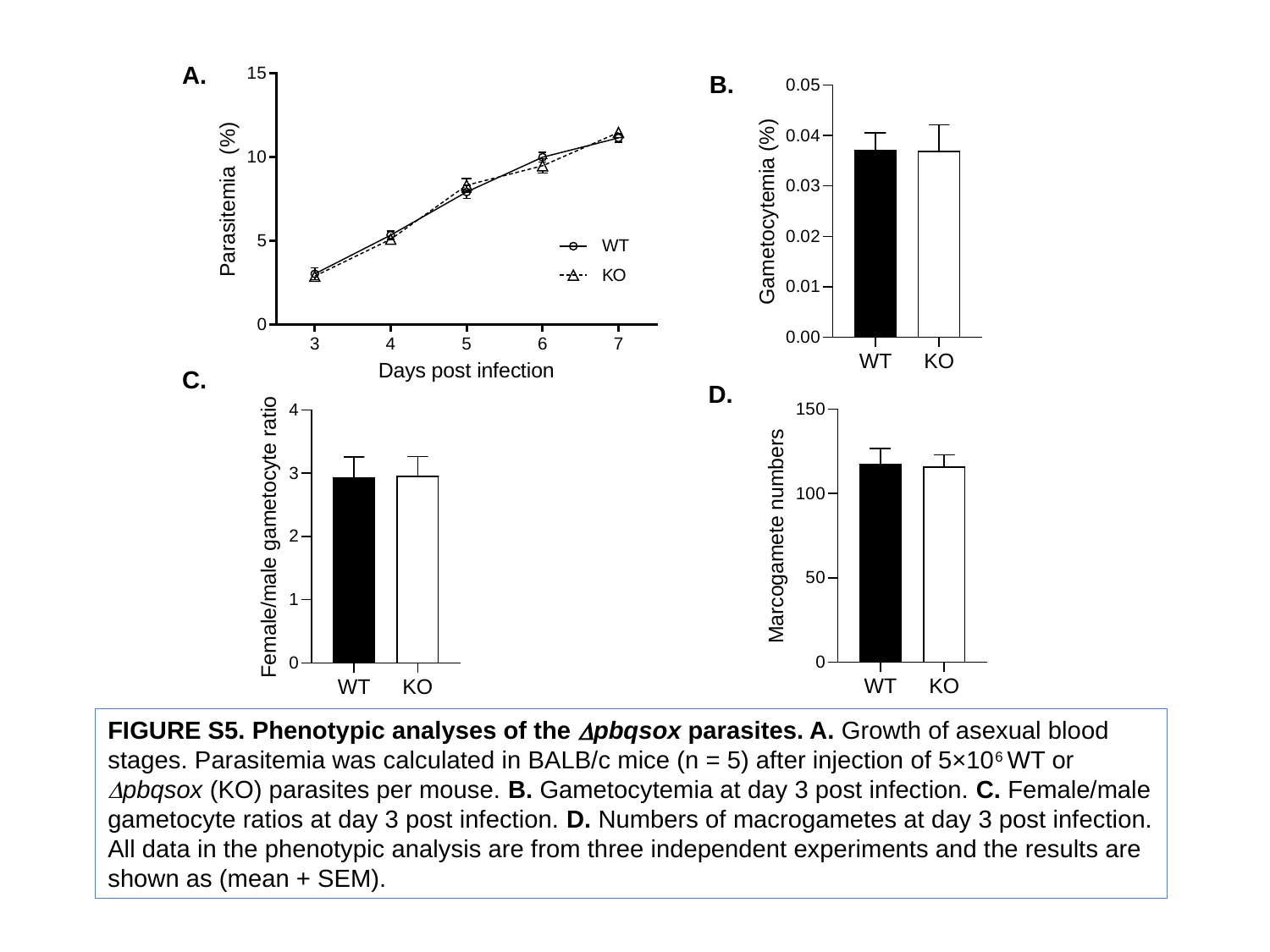

A.
B.
C.
D.
FIGURE S5. Phenotypic analyses of the Δpbqsox parasites. A. Growth of asexual blood stages. Parasitemia was calculated in BALB/c mice (n = 5) after injection of 5×10^6^ WT or Δpbqsox (KO) parasites per mouse. B. Gametocytemia at day 3 post infection. C. Female/male gametocyte ratios at day 3 post infection. D. Numbers of macrogametes at day 3 post infection. All data in the phenotypic analysis are from three independent experiments and the results are shown as (mean + SEM).

## Slide 9
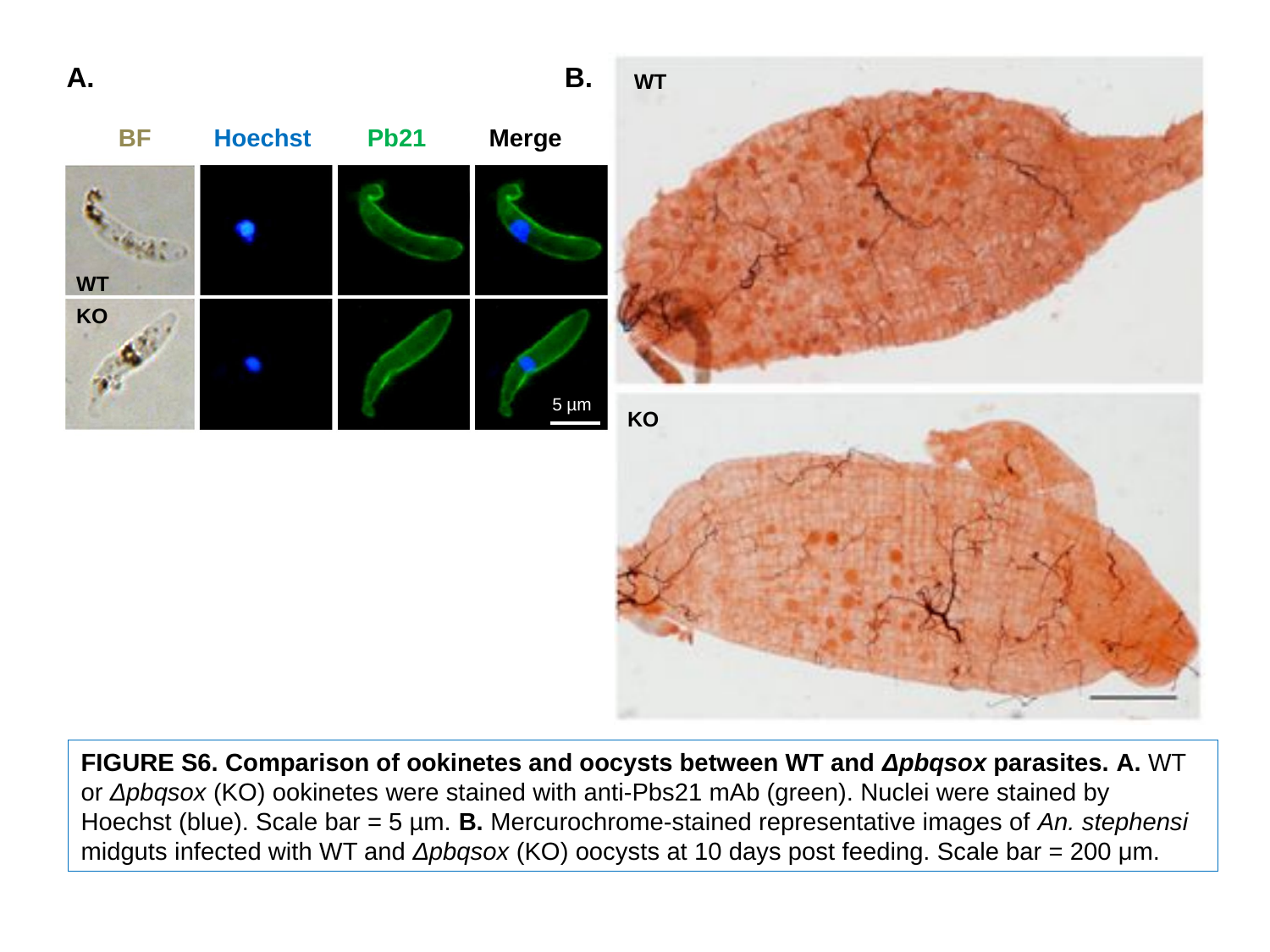

B.
A.
WT
BF Hoechst Pb21 Merge
WT
KO
 WT KO
5 µm
KO
FIGURE S6. Comparison of ookinetes and oocysts between WT and Δpbqsox parasites. A. WT or Δpbqsox (KO) ookinetes were stained with anti-Pbs21 mAb (green). Nuclei were stained by Hoechst (blue). Scale bar = 5 µm. B. Mercurochrome-stained representative images of An. stephensi midguts infected with WT and Δpbqsox (KO) oocysts at 10 days post feeding. Scale bar = 200 μm.
